# A STUB1 ubiquitin ligase/CHIC2 protein complex negatively regulates the IL-3, IL-5, and GM-CSF cytokine receptor common β chain (CSF2RB) protein stability

**DOI:** 10.1016/j.jbc.2022.102484

**Published:** 2022-09-13

**Authors:** Sebastian H.J. Koochaki, Mikołaj Słabicki, Ryan Lumpkin, Charles Zou, Roger Belizaire, Eric S. Fischer, Benjamin L. Ebert

**Affiliations:** 1 Broad Institute of Massachusetts Institute of Technology and Harvard University, Cambridge, Massachusetts, USA; 2 Department of Medical Oncology, Dana-Farber Cancer Institute, Boston, Massachusetts, USA; 3 Harvard-MIT MD/PhD Program, Harvard Medical School, Boston, Massachusetts, USA; 4 Department of Cancer Biology, Dana-Farber Cancer Institute, Boston, Massachusetts, USA; 5 Department of Biological Chemistry and Molecular Pharmacology, Harvard Medical School, Boston, Massachusetts, USA; 6 Howard Hughes Medical Institute, Dana-Farber Cancer Institute, Boston, Massachusetts, USA

**Keywords:** STUB1, CHIC2, CSF2RB, protein degradation, ubiquitin, cell surface receptor, IL-3, cytokine, AML, acute myeloid leukemia, BCA, bicinchoninic acid, cDNA, complementary DNA, FBS, fetal bovine serum, gDNA, genomic DNA, IP, immunoprecipitation, MFI, mean fluorescence intensity, MS, mass spectrometry, RT-qPCR, real-time quantitative PCR, sgRNA, single-guide RNA, TPR, tetratricopeptide repeat

## Abstract

The IL-3, IL-5, and GM-CSF family of cytokines play an essential role in the growth, differentiation, and effector functions of multiple hematopoietic cell types. Receptors in this family are composed of cytokine-specific α chains and a common β chain (CSF2RB), responsible for the majority of downstream signaling. CSF2RB abundance and stability influence the magnitude of the cellular response to cytokine stimulation, but the exact mechanisms of regulation are not well understood. Here, we use genetic screens in multiple cellular contexts and cytokine conditions to identify STUB1, an E3 ubiquitin ligase, and CHIC2 as regulators of CSF2RB ubiquitination and protein stability. We demonstrate that Stub1 and Chic2 form a complex that binds Csf2rb and that genetic inactivation of either *Stub1* or *Chic2* leads to reduced ubiquitination of Csf2rb. The effects of Stub1 and Chic2 on Csf2rb were greatest at reduced cytokine concentrations, suggesting that Stub1/Chic2-mediated regulation of Csf2rb is a mechanism of reducing cell surface accumulation when cytokine levels are low. Our study uncovers a mechanism of CSF2RB regulation through ubiquitination and lysosomal degradation and describes a role for CHIC2 in the regulation of a cytokine receptor.

Cytokine receptors convert extracellular signals into the activation of intracellular pathways that drive proliferation, differentiation, and effector functions of cells. The duration and magnitude of signaling are tightly regulated, and the modulation of cell surface receptor expression is a key mechanism to tune a cell’s response to extracellular signals. For example, CBL is a ubiquitin ligase that targets receptor tyrosine kinases for degradation following activation in order to terminate signaling, and mutations in the RING ubiquitin ligase domain lead to constitutive signaling that drives myeloid malignancies (1). Recent findings have also highlighted the importance of regulation of receptor expression that is independent of receptor activity to attenuate responses to extracellular signals. MARCH1, an E3 ubiquitin ligase, has been shown to modulate the basal gain of the insulin receptor by directly controlling insulin receptor cell surface expression (2). Elucidating both the activity- and nonactivity-dependent mechanisms by which receptor levels are controlled is essential to our understanding of normal cellular homeostasis and the pathogenesis of diseases arising from aberrant signaling.

The IL-3, IL-5, and GM-CSF family of cytokines and respective cytokine receptors play a key role in the growth, differentiation, and effector functions of multiple hematopoietic cell types. Collectively, these cytokines provide a mechanism of communication between adaptive and innate immune cells, as they are primarily produced by activated T cells and stimulate myeloid cells at multiple stages of differentiation (3). The IL-3, IL-5, and GM-CSF receptors are composed of cytokine-specific α chains (IL3RA, IL5RA, and CSF2RA) and a common β chain (CSF2RB). In this class of cytokine receptors, the α chain determines the cytokine specificity, while CSF2RB is responsible for the majority of the signaling downstream of the receptor through the JAK/STAT, PI3K/AKT, and RAS/MAPK pathways (3).

Aberrant signaling downstream of IL-3/IL-5/GM-CSF receptors has been implicated in multiple human diseases including chronic inflammatory diseases and hematologic malignancies (4). GM-CSF receptors are expressed on 80% to 90% of acute myeloid leukemia (AML) patient samples (5, 6, 7), and there is evidence that CSF2RB is constitutively phosphorylated at S585 in AML (8). An activating mutation in CSF2RB has been identified in a case of T-cell acute lymphoblastic leukemia (9). This mutation in the transmembrane domain, R461C, leads to increased CSF2RB protein stability and enhanced cytokine sensitivity in a BaF3 cell line model (9). This suggests that the control of cell surface receptor levels of CSF2RB determines the sensitivity to cytokines and may contribute to the pathogenesis of hematologic malignancies.

The activity- and nonactivity-dependent mechanisms of CSF2RB regulation remain poorly characterized. It has been proposed that ubiquitination, partial proteasomal degradation, endocytosis, and further lysosomal degradation are a possible pathway for CSF2RB degradation (10). However, the ubiquitin ligases involved in the ubiquitination of CSF2RB have not been fully elucidated. It has been previously reported that activated CSF2RB can be negatively regulated by a CUL5^CISH^ complex, although direct ubiquitination has not been demonstrated (11). It has also been hypothesized that CBL may negatively regulate CSF2RB-mediated signaling, but recent evidence suggests that CBL specifically ubiquitinates JAK2 and not CSF2RB directly (12). Here, we employ CRISPR/Cas9 screens to identify the regulators of CSF2RB protein levels in an unbiased manner.

## Results

### CSF2RB is regulated by the ubiquitin-proteasome system

**Figure 1:**
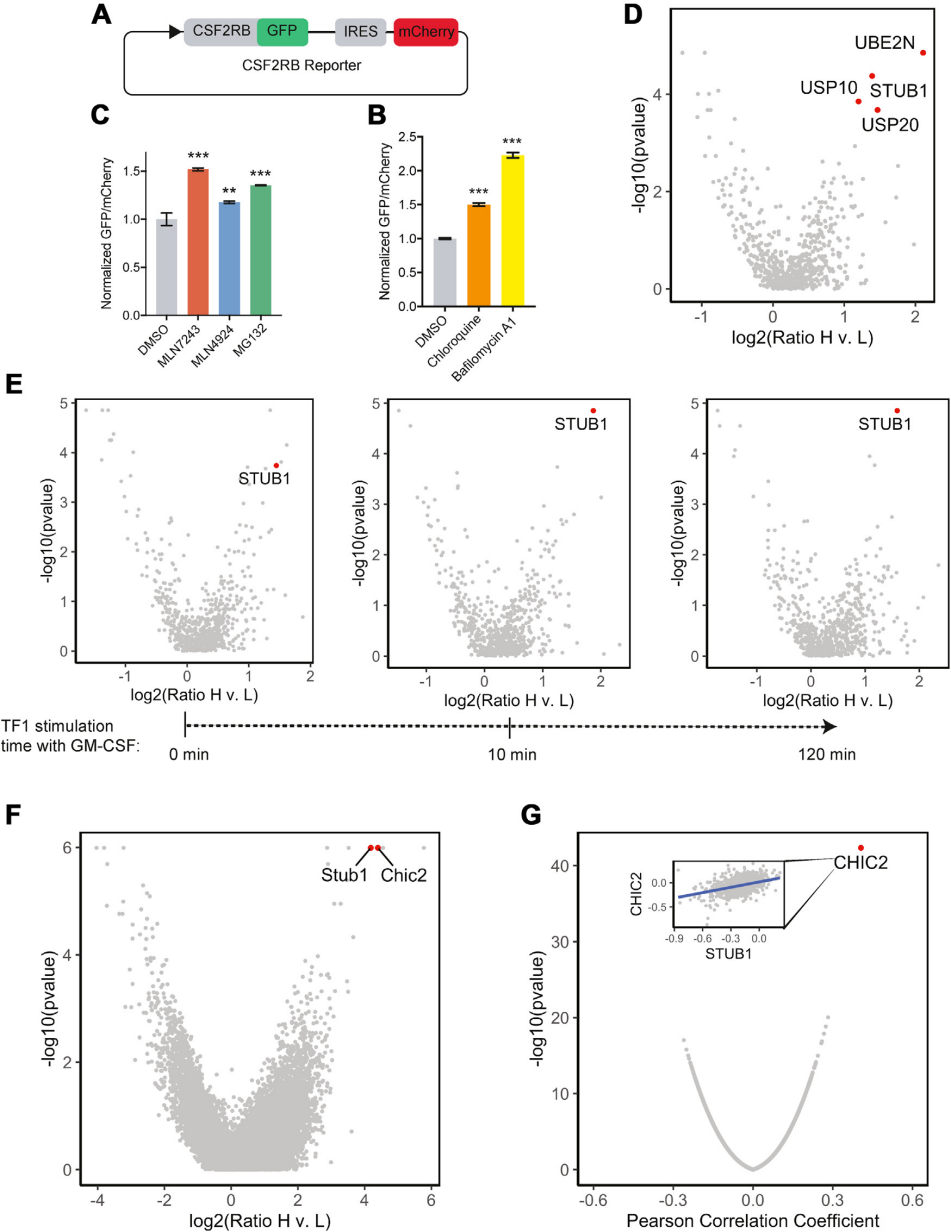
**Ubiquitin ligase–specific and whole-genome CRISPR-Cas9 screens identify STUB1 and CHIC2 as regulators of CSF2RB protein stability in TF1 and 32D cell lines.***A*, diagram of CSF2RB reporter. *B*, bar graph showing normalized GFP/mCherry ratio of CSF2RB reporter in TF1 Cas9 cells treated with DMSO control, 1 μM MLN7243 (E1 inhibitor), 5 μM MLN4924 (neddylation inhibitor), or 10 μM MG132 (proteasomal inhibitor) for 4 h in 5 ng/ml GM-CSF as measured by flow cytometry. Bars are the mean ± SD normalized to the DMSO sample from n = 3 replicates. *p*-values calculated by unpaired Student’s *t* test between DMSO and other conditions. *C*, bar graph showing normalized GFP/mCherry ratio of CSF2RB reporter in TF1 Cas9 cells treated with 10 μM Chloroquine, 100 nM Bafilomycin A1, or a DMSO control for 4 h as measured by flow cytometry. Bars are the mean ± SD normalized to the DMSO sample from three biological replicates. *p*-values calculated by unpaired Student’s *t* test between DMSO and other conditions. *D*, volcano plot showing gene level analysis of CSF2RB reporter-based ubiquitin ligase–specific CRISPR screen in TF1 cells cultured in 5 ng/ml GM-CSF. Guide counts were collapsed to gene level (n = 4 guides/gene; two-sided empirical rank sum test statistics). *E*, volcano plot showing gene level analysis of CSF2RB reporter-based ubiquitin ligase–specific CRISPR screen in TF1 cells starved (0 min) and then stimulated with 5 ng/ml GM-CSF for 10 and 120 min. Guide counts were collapsed to gene level (n = 4 guides/gene; two-sided empirical rank-sum test statistics). *F*, volcano plot showing gene level analysis of Csf2rb reporter-based whole-genome CRISPR screen in 32D cells cultured in 0.01 ng/ml IL-3. Guide counts were collapsed to gene level (n = 4 guides/gene; two-sided empirical rank-sum test statistics). *G*, volcano plot of Pearson correlation coefficient and -log10(*p*-value) for linear correlation analysis of all gene CERES scores with *STUB1* CERES scores in the Cancer Dependency Map (21Q4 public dataset). Inset shows scatterplot of CERES scores for STUB1 (horizontal axis) and CHIC2 (vertical axis).DMSO, dimethyl sulfoxide.

To identify regulators of CSF2RB protein levels, we employed a protein stability reporter system which allows for the direct, quantitative measurement of CSF2RB protein levels in live cells. In this protein reporter system, CSF2RB is fused in-frame to GFP, and an IRES-mCherry is utilized as a transcriptional control (Fig. 1*A*) (12, 13). Measurement of the GFP/mCherry fluorescence ratio by flow cytometry allows for quantitative assessment of CSF2RB protein levels on a single cell level.

We expressed this human CSF2RB reporter in two cell lines: TF1 cells, a human, GM-CSF-dependent, myeloid cell line and 32D cells, a murine, IL-3-dependent, myeloid cell line. To test whether CSF2RB is regulated by the ubiquitin-proteasome system, we utilized small molecule inhibitors of E1 ubiquitin–activating enzymes (MLN7243), NEDD8-activating enzymes (MLN4924), and the proteasome (MG132). In both 32D and TF1 cells, E1 and proteasomal inhibition led to an increase in the CSF2RB GFP/mCherry ratio, while inhibition of neddylation, an essential modification for cullin-RING ubiquitin ligase activation, had minimal effect (Figs. 1*B* and S1*C*). Similar results were found for endogenous CSF2RB protein levels (Fig. S1*A* and S1*D*). We also found that lysosomal acidification inhibition by Bafilomycin A1 led to increased CSF2RB reporter levels and endogenous protein levels in both cell lines, suggesting that lysosomal degradation also plays a role in CSF2RB regulation (Fig. 1*C*, S1*B*, S1*E*, and S1*F*). In summary, our results indicate that in both human and murine cells, CSF2RB/Csf2rb is likely regulated by a noncullin ubiquitin ligase and lysosomal acidification.

### A genetic screen identifies STUB1 as a negative regulator of CSF2RB stability

To gain insight into the molecular machinery required for the ubiquitination and degradation of CSF2RB, we employed a CRISPR/Cas9 screen using the CSF2RB reporter and a previously described ubiquitin ligase–specific single-guide RNA (sgRNA) library (14) in two human leukemia cell lines that endogenously respond to GM-CSF, TF1, and THP1 cells. Cells were starved, acutely stimulated, or chronically stimulated with GM-CSF prior to cell sorting (schematic summary shown in Fig. S2). Cells with the top 5% and bottom 5% GFP/mCherry ratio were isolated using fluorescence-activated cell sorting, and the relative sgRNA abundance in the GFP/mCherry high and low populations were compared.

Analysis of the genetic screens revealed that sgRNA targeting *STUB1* was significantly enriched in the GFP/mCherry high population across multiple cell lines, regardless of cytokine conditions, indicating that STUB1 may destabilize CSF2RB. In TF1 cells cultured chronically in 5 ng/ml GM-CSF, sgRNAs targeting *STUB1*, *UBE2N*, UPS10, and UPS20 were significantly enriched in cells with stabilized CSF2RB (Fig. 1*D*). Surprisingly, across both cell lines and the varied cytokine conditions (starved, acutely stimulated, and chronically stimulated), *STUB1* was the only consistently significant hit (Figs. 1*E* and S3). In summary, our ubiquitin ligase–specific genetic screens identified STUB1 as a robust regulator of CSF2RB reporter levels in multiple cellular contexts.

### A genome-wide CRISPR screen and codependency analysis identify CHIC2 as a partner of STUB1

While our ubiquitin ligase–specific CRISPR screens identified STUB1 as a hit across multiple cellular contexts, STUB1, like other ubiquitin ligases, is thought to work in concert with other proteins to interact with and ubiquitinate substrates (15). To identify other partners that could work in concert with STUB1 to regulate CSF2RB stability, we repeated our reporter-based CRISPR screen strategy in an IL-3-dependent murine cell line, 32D, with a genome-wide sgRNA library (Brie) and a murine Csf2rb reporter (Fig. 1*F*). *Stub1* was again one of the most significantly enriched genes in this third cell line, and interestingly, no other known interactor of STUB1 was significantly enriched.

To prioritize other potential hits from our genome-wide screen, we also employed a codependency analysis of the Cancer Dependency Map, a database of genome-wide CRISPR screens across 1054 cancer cell lines, which allows for the identification gene and protein level networks (16, 17, 18). The most significant gene correlation with *STUB1* identified was *CHIC2* (inset of Fig. 1*G*), a poorly characterized protein with no known cellular function. Consistent with the codependency analysis, *Chic2* behaved similarly to *Stub1* in our genome-wide, reporter-based screen (Fig. 1*F*). This correlation and our genome-wide screen suggest that STUB1 and CHIC2 act in concert to regulate CSF2RB levels.

### *Stub1* or *Chic2* KO increases endogenous Csf2rb protein levels and decreases the rate of Csf2rb destruction

**Figure 2:**
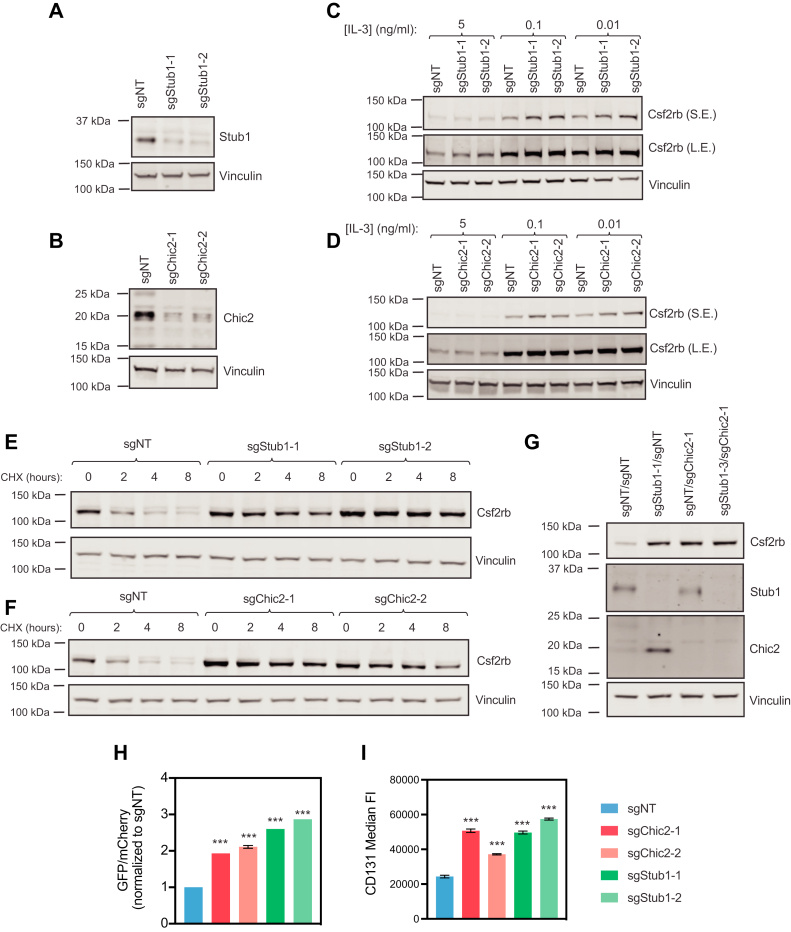
***Stub1* and *Chic2* KO lead to increased endogenous total and cell surface Csf2rb.***A*, Western blots of 32D Cas9 cells with sgNT, sgStub1-1, or sgStub1-2 for STUB1 and Vinculin. *B*, Western blots of 32D Cas9 cells with sgNT, sgChic2-1, or sgChic2-2 for Chic2 and Vinculin. *C*, Western blots of Csf2rb and Vinculin in 32D Cas9 cells with sgNT, sgStub1-1, or sgStub1-2 cultured in 5, 0.1, or 0.01 ng/ml IL-3 (S.E. = short exposure, L.E. = long exposure). Representative of three independent biological replicates with similar results. *D*, Western blots of Csf2rb and Vinculin in 32D Cas9 cells with sgNT, sgChic2-1, or sgChic2-2 cultured in 5, 0.1, or 0.01 ng/ml IL-3. Representative of three independent biological replicates with similar results. *E*, Western blots of Csf2rb and Vinculin in 32D Cas9 cells with sgNT, sgStub1-1, or sgStub1-2 treated with 10 μM cycloheximide (CHX) for 0, 2, 4, or 8 h. *F*, Western blots of Csf2rb and Vinculin in 32D Cas9 cells with sgNT, sgChic2-1, or sgChic2-2 treated with 10 μM cycloheximide (CHX) for 0, 2, 4, or 8 h. *G*, Western blots of Csf2rb, Stub1, Chic2, and Vinculin in 32D Cas9 cells with sgNT/sgNT, sgStub1-1/sgNT, sgNT/sgChic2-1, or sgStub1-1/sgChic2-2. *H*, bar graph showing ratio of mean GFP to mean mCherry signal of Csf2rb reporter in 32D cells with sgNT, sgChic2-1/2, or sgStub1-1/2 in 0.01 ng/ml IL-3 as measured by flow cytometry. Bars show GFP/mCherry ± SD from n = 3 replicates. *I*, bar graph showing median fluorescence intensity for anti-Csf2rb-PE (CD131) in 32D Cas9 cells with sgNT, sgChic2-1/2, or sgStub1-1/2 in 0.01 ng/m IL-3 as measured by flow cytometry. Bars show mean ± SD from n = 3 replicates. *p*-values calculated by unpaired Student’s *t* test between sgNT and other conditions at each cytokine concentration.

Having identified a role for STUB1 and CHIC2 in regulating the CSF2RB reporter, we next sought to examine the effect of these proteins on endogenous CSF2RB. We generated 32D cells with CRISPR/Cas9 inactivation of *Stub1* and *Chic2* with two independent sgRNAs each (Fig. 2, *A* and *B*). Endogenous Csf2rb protein levels were increased with *Stub1* and *Chic2* KO, especially at lower concentrations of IL-3 (Fig. 2, *C* and *D*). Quantification of Western blot replicates at 0.01 ng/ml IL-3 demonstrated a 2- to 3-fold increase in Csf2rb protein levels with *Stub1* or *Chic2* KO (Fig. S4*A* and S4*B*). To test whether this increase in endogenous Csf2rb protein expression was not due to an increase in mRNA transcription, we examined *Csf2rb* mRNA expression by quantitative RT-PCR. Expression of *Csf2rb* mRNA was not increased with *Stub1* or *Chic2* KO, consistent with posttranslational regulation of Csf2rb by Stub1 or Chic2 (Fig. S4*C* and S4*D*).

We next sought to test whether this effect on Csf2rb was indeed on protein stability with a cycloheximide-chase experiment. KO of *Stub1* or *Chic2* reduced the rate of Csf2rb steady-state destruction with cycloheximide treatment over an 8 h time course (Fig. 2, *E* and *F*). Therefore, Stub1 and Chic2 directly regulate Csf2rb protein stability.

### Double KO of *Stub1* and *Chic2* do not have an additive effect on Csf2rb stability

Given that both *Stub1* and *Chic2* KO led to increased protein stability, we next sought to test whether *Stub1* and *Chic2* KO together leads to an additive effect on Csf2rb stability. We generated 32D Cas9 cells with *Stub1* KO*, Chic2* KO, and both *Stub1*/*Chic2* double KO and measured Csf2rb protein levels by Western blot. *Stub1* and *Chic2* KO led to an equal increase in Csf2rb protein levels, and the *Stub1*/*Chic2* double KO did not increase Csf2rb stability beyond the single KOs alone (Fig. 2*G*). Of note, *Stub1* KO led to increased Chic2 protein levels, while *Chic2* KO did not lead to increased Stub1 protein levels, suggesting that Chic2 may be consumed in Stub1’s mechanism of Csf2rb regulation. Taken together, this further supports the hypothesis that Stub1 and Chic2 likely regulate Csf2rb protein stability by the same mechanism and in the same pathway.

### *Stub1* or *Chic2* KO increases endogenous cell surface Csf2rb levels

Having found that *Stub1* and *Chic2* KO led to increased total protein levels of Csf2rb, we hypothesized that *Stub1* and *Chic2* KO specifically lead to cell surface accumulation of Csf2rb, where it is able to participate in active signaling. As measured by flow cytometry, both endogenous, cell surface Csf2rb and a Csf2rb reporter were both increased ∼2.5- to 3-fold with *Stub1* and *Chic2* KO in 0.01 ng/ml IL-3 (Fig. 2, *H* and *I*, representative flow plots for 2I in Fig. S5). We did not see any change in cell surface Il3ra expression as measured by flow cytometry with either *Stub1* or *Chic2* KO, regardless of IL-3 concentration (Fig. S6). Therefore, *Stub1* and *Chic2* KO lead to increased total Csf2rb protein levels and cell surface protein levels at low concentrations of IL-3.

### STUB1 and CHIC2 coimmunoprecipitate

**Figure 3:**
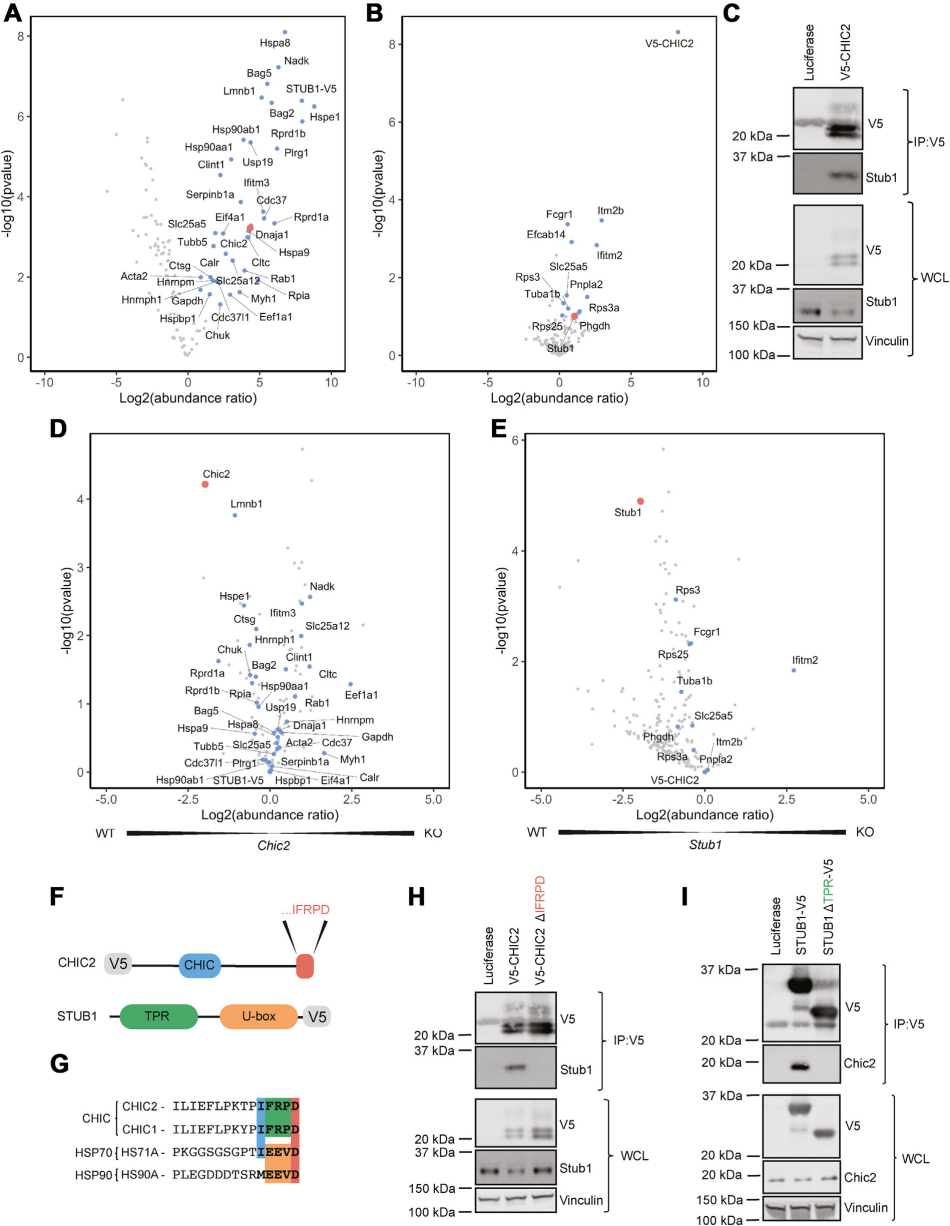
**IP/MS of STUB1 and CHIC2 reveals that STUB1 and CHIC2 interact, and further analysis shows that the TPR domain of STUB1 and C terminus of CHIC2 are required for the interaction.***A*, volcano plot of STUB1-V5 IP comparing STUB1 IP in *Chic2* WT 32D cells to Luciferase control IP from n = 4 replicates. Labeled datapoints have *p*-value < 0.01, and CHIC2 is highlighted in *red*. *B*, volcano plot of CHIC2-V5 IP comparing CHIC2 IP in *Stub1* WT 32D cells to Luciferase control IP from n = 4 replicates. Labeled datapoints have *p*-value < 0.1, and STUB1 is highlighted in *red*. *C*, Western blots of anti-V5 IP of V5-CHIC2 and whole cell lysates for V5, Stub1, and Vinculin from 32D Cas9 cells cultured in 0.01 ng/ml IL-3. *D*, volcano plot of STUB1 IP in *Chic2* WT 32D cells compared to STUB1 IP in *Chic2* KO 32D cells from n = 4 replicates. Labeled datapoints are those with *p*-value < 0.01 from STUB1 IP in *Chic2* WT cells compared to Luciferase control (Fig. 4*B*), and CHIC2 is highlighted in *red*. *E*, volcano plot of CHIC2 IP in *Stub1* WT 32D cells compared to CHIC2 IP in *Stub1* KO 32D cells from n = 4 replicates. Labeled datapoints are those with *p*-value < 0.1 from CHIC2 IP in *Stub1* WT cells compared to Luciferase control (Fig. 4*C*), and STUB1 is highlighted in *red*. *F*, diagram of known CHIC2 and STUB1 protein domains. *G*, amino acid sequences of the C-terminal 15 amino acids of CHIC family members (CHIC1, CHIC2), HSP70 family members (HS71A), and HSP90 family members (HS90A). *H*, Western blots of anti-V5 IP of V5-CHIC2 or V5-CHIC2 ΔIFRPD and whole cell lysate for V5, Stub1, and Vinculin from 32D Cas9 cells cultured in 0.01 ng/ml IL-3. *I*, Western blots of anti-V5 IP of STUB1-V5 or STUB1 ΔTPR-V5 and whole cell lysate for V5, Chic2, and Vinculin from 32D Cas9 cells cultured in 0.01 ng/ml IL-3. IP, immunoprecipitation; MS, mass spectrometry.

To better understand the molecular components of the STUB1/CHIC2 complex in cells, we employed V5-immunoprecipitation (IP) of overexpressed V5-tagged STUB1 or CHIC2 in 32D cells followed by mass spectrometry (MS). In the STUB1 IP/MS, we found that Chic2 and multiple known partners of STUB1, including heat shock proteins, were significantly enriched (Fig. 3*A*). In the CHIC2 IP/MS, we likewise found that Stub1 was enriched, although not significantly, as well as Itm2b, Ifitm2, and Fcgr1 (Fig. 3*B*). Importantly, no interactions were identified between CHIC2 and heat shock proteins, suggesting that heat shock protein/STUB1 complexes are independent of the CHIC2-containing complexes. Furthermore, there were no common interactors identified between the STUB1 and CHIC2 IP/MS experiments, suggesting that the STUB1/CHIC2 complex may not have other components. In validation experiments, we found that V5-CHIC2 coimmunoprecipitated with endogenous Stub1 by IP-Western blot, demonstrating that STUB1 and CHIC2 do indeed interact (Fig. 3*C*). We also found that STUB1-V5 coimmunoprecipitated with endogenous Chic2 and that different concentrations of IL-3 did not alter this interaction (Fig. S7)

To better understand the effects of CHIC2 on the STUB1 interactome and *vice versa*, we preformed a STUB1 IP in *Chic2* KO 32D cells and a CHIC2 IP in a *Stub1* KO 32D cells, each followed by MS (Fig. 3, *D* and *E*). Few STUB1 interactors were significantly lost (*p*-value < 0.01) with *Chic2* KO. These included Lmnb1 and Hspe1, but these proteins are thought to be located on the nuclear membrane and mitochondria, respectively, and not likely to play a role in cell surface regulation of Csf2rb (19). CHIC2 IP in *Stub1* KO cells resulted in the loss of only RPS13/RPS25, ribosomal proteins, and FCGR1, an IgG receptor, were significantly lost with *Stub1* KO. Taken together, these IP/MS experiments suggest that STUB1 and CHIC2 interact but do not have common binding partners and do not significantly alter the interactome of each other.

### CHIC2 interacts with the tetratricopeptide repeat domain of STUB1 *via* its C terminus

We next sought to determine the domains of STUB1 and CHIC2 required for the interaction with each other. A previous study using computational modeling identified putative interactions between the tetratricopeptide repeat (TPR) domain of STUB1, a common structural motif known to mediate protein–protein interactions, and the C terminus of proteins predicted an interaction between STUB1’s TPR domain and the last five amino acids on the C terminus of CHIC2 (Fig. 3*F*) (20). Compared to known STUB1 binding partners, HSP70 and HSP90 family members (*e.g*., HS71A and HS90A), the C-terminal amino acid sequences of CHIC1/CHIC2 are similar but distinct (Fig. 3*G*). Due to these differences, Ravalin *et al*., suggest that the CHIC1/CHIC2 C terminus may participate in a higher affinity interaction with STUB1 compared to HSP70 or HSP90 family members (21).

Based on this modeling data, we hypothesized that the interaction between STUB1 and CHIC2 is dependent on STUB1’s TPR domain and CHIC2’s C terminus. We found that the co-IP of endogenous Stub1 with V5-CHIC2 was lost when the last five amino acids of CHIC2 (ΔIFRPD) were deleted (Fig. 3*H*). Furthermore, we found that co-IP of endogenous Chic2 with STUB1-V5 was lost when we deleted the TPR domain of STUB1 (Fig. 3*I*). Taken together, our data indicate that CHIC2 and STUB1 interact and that both CHIC2’s C terminus and STUB1’s TPR domain are required for their interaction.

### Inhibition of HSP70 or HSP90 family members does not alter Csf2rb protein stability

Given that STUB1 is known to function with multiple HSP family members and that HSP family members were not identified in our genetic screens or IP/MS analyses of CHIC2, we sought to directly test whether inhibition of HSP family members had any effect on CSF2RB protein stability. Since there is significant redundancy among HSP family members, we utilized small molecule inhibitors of HSPs, including Apoptazole, to inhibit HSP70 proteins and HSP990 to inhibit HSP90 proteins. Treatment of 32D cells with either inhibitor did not significantly increase Csf2rb protein levels, suggesting that HSP proteins likely do not play a role in the Stub1/Chic2 regulation of Csf2rb (Fig. S8).

### The Stub1/Chic2 complex interacts with Csf2rb

**Figure 4:**
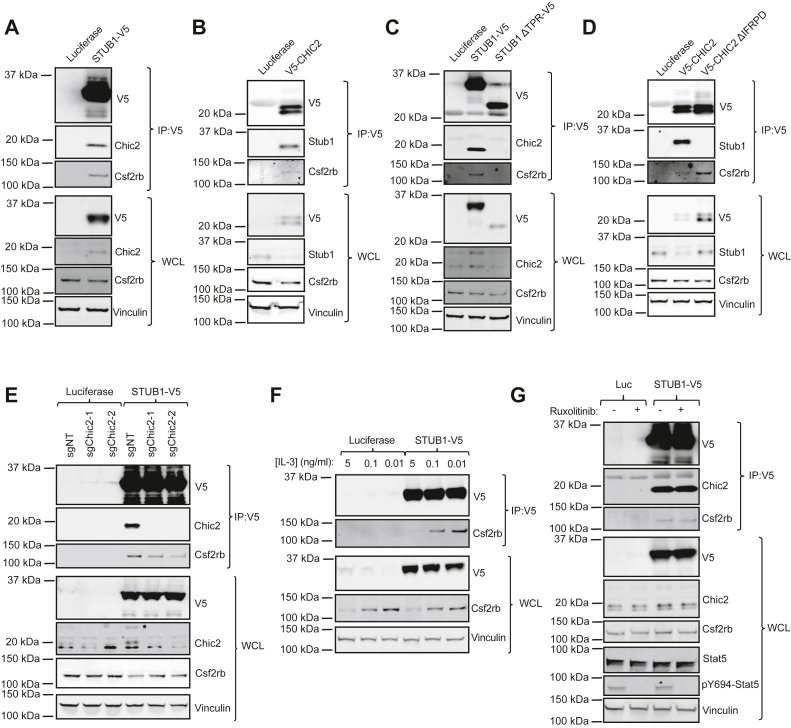
**The Stub1/Chic2 complex interacts with Csf2rb and *Chic2* and *Stub1* KO reduce Csf2rb ubiquitination.***A*, Western blots of anti-V5 immunoprecipitation of STUB1-V5 and whole cell lysate for V5, Chic2, Csf2rb, and Vinculin from 32D Cas9 cells cultured in 0.01 ng/ml IL-3. *B*, Western blots of anti-V5 immunoprecipitation of V5-CHIC2 and whole cell lysate for V5, Stub1, Csf2rb, and Vinculin from 32D Cas9 cells cultured in 0.01 ng/ml IL-3. *C*, Western blots of anti-V5 immunoprecipitation of STUB1-V5 and whole cell lysate for V5, Csf2rb, and Vinculin from 32D Cas9 cells cultured in 5, 0.1, or 0.01 ng/ml IL-3. *D*, Western blots of anti-V5 immunoprecipitation of STUB1-V5 or STUB1 ΔTPR-V5 and whole cell lysate for V5, Csf2rb, Chic2, and Vinculin from 32D Cas9 cells cultured in 0.01 ng/ml IL-3. *E*, Western blots of anti-V5 immunoprecipitation of STUB1-V5 and whole cell lysate for V5, Csf2rb, Chic2, and Vinculin in 32D Cas9 cells with sgNT or sgChic2-1/2 cultured in 0.01 ng/ml IL-3. *F*, Western blots of anti-V5 immunoprecipitation of STUB1-V5 and whole cell lysate for V5, Csf2rb, and Vinculin from 32D Cas9 cells cultured in 5, 0.1, or 0.01 ng/ml IL-3. *G*, Western blots of anti-V5 immunoprecipitation of STUB1-V5 and whole cell lysate for V5, Csf2rb, Chic2, and Vinculin from 32D Cas9 cells cultured in 0.01 ng/ml IL-3 and treated with or without Ruxolitinib (100 nM) for 2 h.

Because both *Stub1* and *Chic2* KO increased Csf2rb protein stability, we hypothesized that Stub1 and Chic2 interact directly with Csf2rb. We found that both STUB1-V5 and V5-CHIC2 coimmunoprecipitated with endogenous Csf2rb (Fig. 4, *A* and *B*). To explore the components of the STUB1/CHIC2 complex required for the interaction with CSF2RB, we next tested the effect of deletion of the C terminus of CHIC2 and the deletion of the TPR domain of STUB1 on their interaction with Csf2rb. Loss of the TPR domain of STUB1, thereby preventing interaction with Chic2, blocked co-IP of endogenous Csf2rb (Fig. 4*C*). Deletion of the C terminus of CHIC2 (ΔIFRPD) did not affect the co-IP of Csf2rb with V5-CHIC2, while the interaction with Stub1 was again lost (Fig. 4*D*). This suggests that Chic2 binds to Csf2rb independent of Stub1, and we hypothesized that Chic2 may facilitate the interaction between Stub1 and Csf2rb. *Chic2* KO reduced but did not block co-IP of Csf2rb with STUB1-V5 (Fig. 4*E*), suggesting that either Chic2 and Stub1 bind independently of each other to Csf2rb or there are yet other proteins in the complex not captured by our genetic and proteomic techniques.

### Reduced cytokine concentration increases the interaction between Stub1/Chic2 and Csf2rb

To understand the conditions required for the regulation and interaction of Stub1/Chic2 with Csf2rb, we tested the effects of cytokine concentration and receptor activation on the interaction between Csf2rb and Stub1/Chic2. We hypothesized that reduced cytokine concentration would enhance the interaction between Stub1 and Csf2rb. Endogenous Csf2rb co-IP with STUB1-V5 increased with reduced IL-3 concentration (Fig. 4*F*); however, this increased interaction was commensurate with the increased expression of Csf2rb in reduced IL-3 concentration, suggesting that the interaction between Stub1 and Csf2rb is more dependent on concentration of Csf2rb rather than inactivity. Indeed, treatment of 32D cells with Ruxolitinib to inhibit Jak2 phosphorylation of Csf2rb did not alter the interaction between STUB1-V5 and Csf2rb, suggesting that Stub1 likely binds to inactivated and activated Csf2rb equally (Fig. 4*G*).

### KO of *Stub1* or *Chic2* reduces ubiquitination of CSF2RB

**Figure 5:**
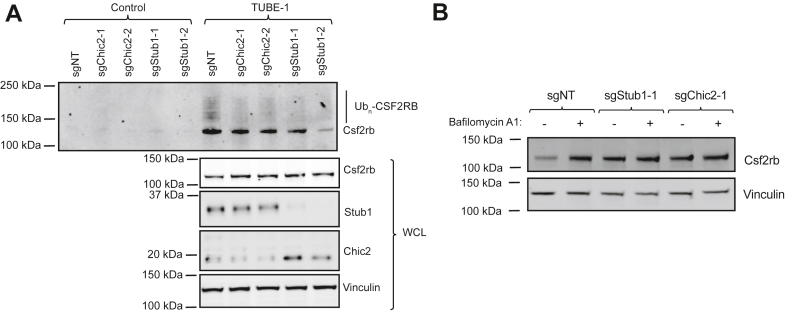
***Stub1* and *Chic2* KO lead to deceased ubiquitination of Csf2rb and blocks the effect of a lysosomal acidification inhibitor on Csf2rb stability.***A*, Western blots of TUBE immunoprecipitation and whole cell lysate for Csf2rb, Chic2, Stub1, and Vinculin as a loading control in 32D Cas9 cells with sgNT, sgChic2-1/2, or sgStub1-1/2 cultured in 0.01 ng/ml IL-3 and treated with 1 μM Bafilomycin A1 and 10 μM MG132 for 4 h. *B*, Western blots of Csf2rb and Vinculin in 32D cas9 cells with sgNT, sgStub1-1, or sgChic2-1 treated with DMSO or bafilomycin A1 (100 nM) for 16 h. DMSO, dimethyl sulfoxide.

Given the effects of *Stub1* and *Chic2* KO on Csf2rb protein stability, we hypothesized that Stub1 and Chic2 are involved in ubiquitination of Csf2rb. To test ubiquitination of endogenous Csf2rb, we employed Tandem Ubiquitin Binding Entity (TUBE) agarose beads, which selectively enrich ubiquitinated proteins. Western blot analysis of TUBE-enriched protein lysates showed that *Stub1* or *Chic2* KO led to reduced ubiquitination of Csf2rb (Fig. 5*A*). Therefore, Chic2 and Stub1 interact with Csf2rb, and while CHIC2 is not required for this interaction, both *Chic2* and *Stub1* KO reduce Csf2rb ubiquitination.

### KO of *Stub1* or *Chic2* blocks the effect of a lysosomal acidification inhibitor (Bafilomycin A1) on Csf2rb protein stability

Given that lysosomal acidification inhibitors increased Csf2rb protein stability, we hypothesized that Stub1 and Chic2 may regulate Csf2rb through lysosomal degradation. To test this, we treated WT and *Stub1* or *Chic2* KO 32D cells with a lysosomal acidification inhibitor, Bafilomycin A1. KO of *Stub1* or *Chic2* blocked the effect of Bafilomycin A1 on Csf2rb stability (Fig. 5*B*). Taken together, our data suggests that Stub1/Chic2 ubiquitinates Csf2rb, leading to lysosomal degradation.

## Discussion

In this study, we used genetics screens, analysis of the Cancer Dependency Map, and IP/MS to discover a mechanism of CSF2RB regulation by a STUB1/CHIC2 complex. We found that *Stub1* and *Chic2* KO lead to increased total and cell surface protein levels of Csf2rb at low cytokine concentrations, and these effects were limited only to the β chain and not the cytokine-specific α chain (Il3ra). Furthermore, we found that Stub1 interacts with Csf2rb at low cytokine concentrations and that *Stub1* and *Chic2* KO reduce ubiquitination of Csf2rb. Finally, *Stub1* and *Chic2* KO abrogated the effect of lysosomal acidification inhibitors on Csf2rb protein stability, suggesting that ubiquitination by Stub1/Chic2 likely leads to lysosomal degradation. These data are consistent with a model in which the STUB1/CHIC2 complex ubiquitinates CSF2RB leading to lysosomal degradation to prevent cell surface accumulation in the absence of cytokine.

STUB1 has been previously found to regulate diverse classes of receptors, both membrane bound and otherwise, including the glucocorticoid receptor, mineralocorticoid receptor, androgen receptor, estrogen receptor, epidermal growth factor receptor (EGFR and ERBB2), MET receptor, insulin receptor, toll-like receptor 4, and the IL-4 receptor (20, 22, 23, 24, 25, 26, 27, 28, 29, 30, 31). Classically, STUB1 is thought to work primarily with HSP70 and HSP90 complexes to ubiquitinate misfolded substrates (15, 32, 33, 34). Our data indicate that STUB1 likely does not require HSP70 or HSP90 to regulate CSF2RB but instead interacts with CHIC2 to regulate receptor levels and activity. Data supporting this conclusion are that (1) HSP inhibitors did not lead to significant changes in Csf2rb protein levels, (2) HSPs were not detected in the CHIC2 IP/MS experiment, and (3) Chic2 occupies the same binding site (TPR domain) on Stub1 as HSPs. Our study therefore highlights the potential importance of an HSP70/HSP90-independent role of STUB1 in the ubiquitination of a substrate.

Our studies also identify a role for CHIC2 in the ubiquitination of a receptor. CHIC2 is a poorly characterized protein with no known cellular function. CHIC2 has primarily been studied in the context of oncogenic fusion proteins, and *CHIC2-ETV6* fusions have been found in rare cases of AML (35). Our study provides a starting point for understanding the cellular function of CHIC2 in STUB1-mediated receptor ubiquitination. While Chic2 was not required for the interaction between Stub1 and Csf2rb, *Chic2* KO reduced ubiquitination of CSF2RB through an unclear mechanism. This suggests that there may be other components of the complex that work with CHIC2 and STUB1 that were not captured by our genetic and proteomic techniques. STUB1 and CHIC2 dependency scores also correlated across cell lines in the Cancer Dependency Map, suggesting that this complex may have wide ranging effects on multiple receptors. Further biochemical and biological characterization of CHIC2 and the STUB1/CHIC2 complex may uncover a role for CHIC2 and other potential complex members in the STUB1-mediated ubiquitination of other receptors.

Finally, our studies illustrate a mechanism of receptor regulation that is not dependent on the activity of the receptor. The effect of STUB1 and CHIC2 on CSF2RB was inversely correlated with cytokine concentration, and treatment with Ruxolitinib to inhibit CSF2RB phosphorylation did not change the interaction between STUB1 and CSF2RB. This is in contrast to other mechanisms of receptor regulation in which a ubiquitin ligase, such as by CBL, primarily ubiquitinates activated receptors following acute cytokine stimulation to terminate signaling. Loss of Stub1 or Chic2 led to increased cell surface levels of Csf2rb at reduced concentrations of cytokine. Therefore, this STUB1/CHIC2 complex appears to regulate the turnover of CSF2RB to decrease cell surface accumulation. In aggregate, our studies discover and characterize a STUB1/CHIC2 complex that controls CSF2RB cell surface receptor levels through ubiquitination and lysosomal degradation.

## Experimental procedures

### Cell culture

32D (ATCC CRL-11346), TF1 (ATCC CRL-2003), and THP1 (ATCC TIB-202) cells were maintained in in RPMI (Corning) with 10% fetal bovine serum (FBS) (Sigma) and 1x penicillin–streptomycin–glutamine supplement (Thermo Fisher Scientific). 32D medium was supplemented with 5 ng/ml recombinant murine IL-3 (PeproTech, #213-13), and TF1 medium was supplemented with 5 ng/ml recombinant human GM-CSF (Miltenyi Biotec, #130-093-866). HEK293T cells (ATCC CRL-3216) for lentiviral production were maintained in Dulbecco's modified Eagle's medium (Corning) with 10% FBS. All cell lines were maintained at 37 °C and 5% CO2.

### Plasmids and cloning

Oligonucleotides (synthesized by Eton Bioscience) used for generating sgRNA constructs are listed in the table later. sgRNA targeting mouse and human *STUB1* (sgStub1-1/2 and sgSTUB1-1/2) and *CHIC2* (sgChic2-1/2 and sgCHIC2-1/2) or a nontargeting sgRNA (sgNT) were cloned into sgRNA expression vectors with fluorophores, U6.sgRNA.SFFV.TagBFP, TagRFP, or RFP657, as per the Zhang lab protocol (36). sgRNA oligonucleotides were phosphorylated with T4 polynucleotide kinase (NEB, M0201S) and cloned into BsmBI-linearized (NEB, R0580), gel purified sgRNA expression vectors using T4 DNA ligase (NEB, M0202). *STUB1* (ccsbBroadEn_02378, NM_005861.3) and *CHIC2* (csbBroadEn_08021, NM_012110.3) complementary DNAs (cDNAs) were obtained from the Broad Institute’s Genomic Perturbation Platform in pDONR223. *CSF2RB* cDNA was obtained from Dharmacon Inc (Catalog OHS5894-202504091, clone 100000054) in pDONR223. Murine *Csf2rb* cloned into the Artichoke backbone (Addgene, plasmid 73320) was synthesized by Twist Biosciences. *STUB1* and *CHIC2* cDNAs were mutagenized with site-directed mutagenesis (NEB Q5 Site-Directed Mutagenesis Kit, E0554) in the pDONR223 plasmid. Oligonucleotides used for *STUB1/CHIC2* cDNA mutagenesis are listed later. Lentiviral expression constructs for modified *STUB1* and *CHIC2* were generated *via* Gateway LR Clonase (Thermo Fisher Scientific, 11791100) reaction between the pDONR223 plasmid and lentiviral destination plasmid pRRL-SFFV-IRES-GFP or -dTomato (kindly provided by Christopher Baum and Axel Schambach, Hannover Medical School, Hannover, Germany). *CSF2RB* in pDONR223 was likewise cloned into a gateway reporter plasmid derived from the Artichoke plasmid (Addgene, plasmid 73320). Cas9 was expressed using pXR101-Cas9 (Addgene, plasmid 52962) or pXPR311-Cas9 (Addgene, plasmid 96924) (also known as pLX311-Cas9). All constructs were confirmed by Sanger sequencing (Eton Biosciences).

### sgRNA sequences

sgChic2-1 (Mouse): ATCTTCCTGTTAATGTGCGG

sgChic2-2 (Mouse): GCACGTTAGGGTGCAGTATG

sgStub1-1 (mouse): GGAGATGGAGAGTTATGATG

sgStub1-2 (mouse): CGTGGGCCGCAAGTACCCGG

sgNT (nontargeting): ACGGAGGCTAAGCGTCGCAA

### Oligonucleotide sequences

Add V5 tag + stop codon to STUB1 (adds V5 tag = GKPIPNPLLGLDST and stop = TGA)

STUB1_add_V5_STOP-F: gctgggcctggatagcacctgaTGCCCAACTTTCTTGTACAAAG

STUB1_add_V5_STOP-R: agcgggttcggaatcggtttgccGTAGTCCTCCACCCAGCC

Delete the TPR domain of STUB1 (deletes AA's 26-127)

STUB1_del_TPR-F: CGGCTGAACTTCGGGGAC

STUB1_del_TPR-R: GCTCGGGCTCTTCTCGGG

Add stop codon to CHIC2 (adds stop codon = TGA)

CHIC2_add_STOP-F: tgaTGCCCAACTTTCTTGTAC

CHIC2_add_STOP-R: ATCTGGTCGAAAAATCGG

Add N-terminal V5 tag to CHIC2 (adds V5 tag = GKPIPNPLLGLDST)

CHIC2_add_Nterm-V5-F: ctgctgggcctggatagcaccGCGGATTTCGACGAAATC

CHIC2_add_Nterm-V5-R: cgggttcggaatcggtttgccCATGCCAACTTTTTTGTAC

Delete the last 5 aa's of CHIC2 (IFRPD) and add stop codon (deletes AA's 161-165)

CHIC2_del_IFRPD-F: aTGCCCAACTTTCTTGTAC

CHIC2_del_IFRPD-R: caCGGTGTCTTTGGTAAAAATTC

### Lentivirus production

Lentivirus was produced in a 10 cm^2^ plate by transient transfection of ∼70% confluent HEK293T cells in 10 ml of media using 63 μl TransIT-LT1 (Mirus, MIR2304) in 600 μl Opti-MEM reduced serum medium (Thermo Fisher Scientific, 31985088) containing 10.5 μg lentiviral expression plasmid, 14 μg psPAX2 packaging plasmid (Addgene, 12260), and 5.25 μg pCMV-VSV-G envelope plasmid (Addgene, 8454). Lentiviral supernatant was harvested at 48 h post-transfection and passed through a 0.45 μm syringe filter (Pall, 4614).

### Cell line generation

For lentiviral transduction, 1-2 × 10^6^ cells in 2 ml of appropriate culture media with 4 μg/ml polybrene (Santa Cruz Biotechnology, 134220) were infected in 6-well plates with 400 μl to 1 ml of lentiviral supernatant. Plates were centrifuged at 1050*g* at 37 °C in an Eppendorf 5910R centrifuge. Cells were then cultured overnight and washed 3x in PBS and then cultured as normal. Cells were transduced with pXR101-Cas9 (THP1) or pXPR311-Cas9 (TF1 and 32D) for stable expression of Cas9 and selected with blasticidin (2 μg/ml for TF1, THP1, and U937 and 10 μg/ml for 32D) (Thermo Fisher Scientific, A1113902) for 7 days starting the day after transduction. Cells transduced with the fluorescent reporter plasmid were selected with 2 μg/ml puromycin (Thermo Fisher Scientific, A1113802) for 3 days starting the day after transduction. Cells transduced with lentiviral constructs containing fluorescent proteins (sgRNA expression vectors and pRRL-SFFV expression vectors) were sorted using a Sony SH800S or MA900 cell sorter.

### Small molecule inhibitors

Relevant cells were treated with 1 μM MLN7243 (ChemieTek, CT-M7243), 5 μM MLN4924 (MedChem Express, HY-70062), 10 μM MG132 (Selleck Chemicals, S2619), 10 μM Chloroquine (Selleck Chemicals, S4157), 100 nM Bafilomycin A1 (Cayman Chemical Company, 11038), 1 μM Apoptozole (Selleck Chemicals, S8365), 100 nM HSP990 (Selleck Chemicals, S7097), or dimethyl sulfoxide (VWR, 97063-136) for the reported amount of time.

## Antibodies

**Table:** Primary antibodies:

Antibody	Use	Recognized species	Source	Catalog #	Species	Dilution
AKT	W	Mouse/human	CST	4685	Rabbit	1:1000
pS473-AKT	W	Mouse/human	CST	4060	Rabbit	1:1000
CSF2RB (IL3RB)	W	Mouse	Abcam	ab86743	Rat	1:1000
CSF2RB (CD131) - PE	F	Mouse	BD Bioscience	559920	Rat	1:200
CHIC2	W	Mouse/human	Sigma Aldrich	SAB2103335	Rabbit	1:1000
ERK 1/2	W	Mouse/human	CST	4696	Rabbit	1:1000
pT202/Y205-ERK 1/2	W	Mouse/human	CST	4370	Rabbit	1:1000
IL3RA (CD123)	F	Mouse	BioLegend	106005	Rat	1:200
Rat IgG1,k isotype control	F	Mouse	BioLegend	554685	Rat	1:200
STAT5	W	Mouse/human	CST	94205	Rabbit	1:1000
pY694-STAT5	W	Mouse/human	CST	4322	Rabbit	1:1000
STUB1 (CHIP)	W	Mouse/human	CST	2080	Rabbit	1:1000
V5 tag	W	N/A	Abcam	ab27671	Mouse	1:1000
V5 tag	W	N/A	CST	13202	Rabbit	1:1000
Vinculin	W	Mouse/human	Sigma Aldrich	V9131	Mouse	1:10,000

**Table:** Secondary antibodies:

Antibody	Source	Catalog #	Dilution used
Goat anti-Rabbit IgG – IRDye 800CW	Li-Cor Biosciences	926–32211	1:10,000
Goat anti-Mouse IgG – IRDye 680LT	Li-Cor Biosciences	926–68020	1:10,000

(W, Western blot; F, flow cytometry; CST, Cell Signaling Technologies; TFS, Thermo Fisher Scientific).

### Flow cytometry assays for CSF2RB and IL3RA reporter

Cells were cultured in relevant conditions in 96-well flat bottom plates. Cells were mixed by pipetting, and a CytoFLEX S flow cytometer (Beckman) was used to directly measure the GFP and mCherry signal from 10^4^ events. FlowJo software (https://www.flowjo.com/, version 10) was used to analyze the data. The data were gated on the mCherry+ cell population to ensure all cells analyzed contained the reporter, and the mean GFP and mCherry signals (mean fluorescence intensity [MFI]) was measured. The reported GFP/mCherry ratio was calculated as the (MFI of GFP)/(MFI of mCherry) and then normalized to the relevant condition.

### CRISPR/Cas9 screens

TF1 Cas9 or THP1 Cas9 cells were infected with the custom, ubiquitin ligase–specific sgRNA library cloned into the lentiGuide-puro plasmid (Addgene, plasmid 52963) (14). Cells were then cultured for 8 days in their relevant cell culture media supplemented with 2 μg/ml puromycin and 2 μg/ml blasticidin to select for sgRNA+ cells, maintain Cas9 expression, and allow for gene KO. On the eighth day (day prior to cell sorting), cells were treated in different conditions described later: TF1 Cas9 cells were cultured in four separate conditions in triplicate: chronic 5 ng/ml GM-CSF, GM-CSF-starved, GM-CSF-starved and stimulated with 5 ng/ml GM-CSF for 10 min, or GM-CSF starved and stimulated with 5 ng/ml GM-CSF for 120 min. TF1 Cas9 cells were either cultured in 5 ng/ml GM-CSF or starved of GM-CSF (but maintained in 10% FBS) overnight. The following day GM-CSF starved TF1 Cas9 cells were stimulated with 5 ng/ml GM-CSF for 10 or 120 min or starvation was continued. THP1 Cas9 cells were cultured in two separate conditions in duplicate: no GM-CSF stimulation or 60 min of 5 ng/ml GM-CSF stimulation. THP1 cells were cultured in their normal media overnight and then either maintained in their normal media or stimulated with 5 ng/ml GM-CSF for 60 min the following day. After the appropriate treatments, cells were harvested and put on ice for sorting. Cells were sorted using a Sony SH800S or SH900 cell sorter. Approximately 2 × 10^5^ cells were sorted from the top 5% GFP/mCherry high population and the bottom 5% GFP/mCherry low population. Sorted cells were pelleted and frozen at −20 °C prior to genomic DNA (gDNA) isolation.

For the whole genome screen, 32D Cas9 cells were infected with the Brie whole genome, murine sgRNA library (Addgene #73632). Infected cells were cultured in 0.1 ng/ml IL-3 RPMI + 10% FBS for 6 days and passaged to maintain library representation at 500x. On the sixth day, cells were washed and cultured in 0.01 ng/ml IL-3 RPMI + 10% FBS overnight prior to sorting. After the appropriate treatments, cells were harvested and put on ice for sorting. Cells were sorted using a Sony SH800S or SH900 cell sorter. Approximately 2 × 10^6^ cells were sorted from the top 10% GFP/mCherry high population and the bottom 10% GFP/mCherry low population in quadruplicate. Sorted cells were pelleted and frozen at −20 °C prior to gDNA isolation.

gDNA from sorted cells was isolated using a direct lysis buffer (1 mM CaCl_2_, 3 mM MgCl_2_, 1 mM EDTA, 1% Triton X-100, 10 mM Tris pH 7.5, 0.2 mg/ml Proteinase K) with incubation for 10 min at 65 °C and 15 min at 95 °C. sgRNA sequences were amplified and barcoded from the gDNA and next generation sequencing library was prepared by a two-step PCR. The sgRNA sequence was amplified in a first PCR reaction with eight staggered forward primers. Twenty microliters of direct lysed cells was mixed with 0.04 U Titanium Taq (Takara Bio 639210), 0.5x Titanium Taq buffer, 800 μM dNTP mix, 200 nM SBS3-Stagger-pXPR003 forward primer, 200 nM SBS12-pXPR003 reverse primer in a 50 μl reaction (cycles: 5 min at 94 °C, 15 × (30 s at 94 °C, 15 s at 58 °C, 30 s at 72 °C), 2 min at 72 °C). Two microliters of the first PCR reaction was used as the template for 15 cycles of the second PCR, where Illumina adapters and barcodes were added (0.04 U Titanium Taq, 1x Titanium Taq buffer, 800 μM dNTP mix, 200 nM P5-SBS3 forward primer, 200 nM P7-barcode-SBS12 reverse primer). An equal amount of all samples was pooled and subjected to preparative agarose electrophoresis followed by gel purification using a QIAquick gel extraction kit (Qiagen, 28704). Eluted DNA was further purified by NaOAc and isopropanol precipitation. Amplified sgRNAs were then sequenced using an Illumina NextSeq.

Methods previously described (14) were used to analyze the relative sgRNA abundance in the GFP/mCherry high population and GFP/mCherry low population and to compute gene level statistics. Raw read counts are shown in Supporting Table 1 and analysis results shown in Supporting Table 2.

### Cancer Dependency Map correlation analysis

DepMap Public 21Q4 data containing the CERES scores for all genes across 1054 cancer cell lines was downloaded from the DepMap portal (https://depmap.org/portal/). R statistical software (https://www.r-project.org, version 3.3.1) was used to calculate the Pearson correlation coefficient and *p*-value for correlations between the CERES scores for *STUB1* and the CERES scores for every other gene.

### Real-time quantitative PCR

Total RNA was isolated from samples with a RNeasy mini kit (Qiagen, #74104). RNA concentration was measured using a NanoDrop 8000 (ThermoScientific), and SuperScript IV Vilo master mix (Thermo Fisher Scientific, #11756050) was used to generate cDNA from 1 μg of RNA. Multiplexed real-time quantitative PCR (RT-qPCR) was conducted with 250 ng of cDNA, TaqMan gene expression master mix (ThermoFisher Scientific, 4369016), and TaqMan primers from ThermoFisher Scientific, noted later. Four technical replicates and three biological replicates were analyzed for each condition in 20 μl reactions in a 384-well plate. RT-qPCR was executed with an Applied Biosciences QuantStudio 6. C_t_ values were calculated by the QuantStudio 6 software (Applied Biosciences), and the ΔΔC_t_ method was used to calculate the relative expression.

**Table:** RT-qPCR Primers

Primer ID	Target	Species	Dye	Source	Catalog #
Mm00655745_m1	*Csf2rb*	Mouse	FAM-MGB	Thermo Fisher Scientific	4331182
Mm99999915_g1	*Gapdh*	Mouse	VIC-MGB-PL	Thermo Fisher Scientific	4448484

### Competition assays

32D Cas9 cells expressing relevant sgRNA with BFP or RFP were mixed at 1:1 ratio (1 × 10^5^ cells per sgRNA per well, 2 × 10^5^ total cells per well) in a 96-well plate. A Tecan D300e digital dispenser was used to dispense the appropriate amount of 5 μg/ml recombinant mouse IL-3 in dH_2_O + 0.1% Triton X-100 as per the manufacturer’s instructions. Each condition was done in triplicate. On the second day (day 1) and every following 2 to 3 days, cells were passaged 1:10 days into fresh media, and the remaining percentage of BFP+ and RFP+ cells was measured by flow cytometry on a CytoFLEX S flow cytometer (Beckman).

### Cell surface antibody staining

Approximately 2 × 10^5^ 32D cells expressing relevant sgRNA were harvested from culture and washed 2x with PBS. Cells were then stained with relevant 1:200 diluted PE-conjugated antibodies in 100 μl of PBS for 1 h on ice. Following staining, cells were again washed 2x in PBS, and PE signal was measured by flow cytometry on a CytoFLEX S flow cytometer. FlowJo software (https://www.flowjo.com, version 10) was again used to analyze the data and calculate the median fluorescence intensity of the PE signal.

### Immunoblots

Cells were lysed in a NP40-based lysis buffer (150 mM NaCl, 50 mM Tris pH 7.5, 1% NP40, 1x Halt protease and phosphatase inhibitor cocktail (Thermo Fisher Scientific, 78446)) for 15 min on ice. Protein lysates were precleared by centrifuging at 14,800 RPM for 10 min at 4 °C. Protein lysates were harvested, and protein concentration was measured and normalized using the Pierce bicinchoninic acid (BCA) protein assay kit (Thermo Fisher Scientific, 23225). Samples were prepared in NuPAGE LDS sample buffer (Thermo Fisher Scientific, NP0007) and NuPAGE sample reducing agent (Thermo Fisher Scientific, NP0004) and then boiled at 70 °C for 10 min. Samples were then resolved by SDS-PAGE using NuPAGE 4% to 12% Bis-Tris protein gels (Thermo Fisher Scientific, NP0336), and transferred by electrophoresis at 90 to 100V for 2 h on to 0.45 μm nitrocellulose membranes (Life Technologies, LC2001). Membranes were blocked in Odyssey blocking buffer (Licor, 927-50000) for 1 h at room temperature (RT). Membranes were incubated in primary antibodies, detailed below, overnight at 4 °C in Odyssey blocking buffer. Membranes were washed in 1x Tris-buffered saline with Tween-20 (Cell Signaling, 9997) 3x for 5 min at RT and then incubated in secondary antibodies, detailed later, for 1 h at RT in Odyssey blocking buffer. Membranes were again washed 3x for 5 min at RT in 1x Tris-buffered saline with Tween-20 and then visualized using a Li-Cor Odyssey CLx. Western blots were quantified at relevant exposures using ImageJ (https://imagej.nih.gov/ij/).

### IP

Cells were lysed and precleared as aforementioned. Following protein concentration measurement by Pierce BCA and normalization, a small aliquot of protein lysate was saved for whole cell lysate analysis, and 1 to 2 mg of total protein lysate in 500 μl of lysis buffer was incubated with 50 μl of anti-V5 tag magnetic beads slurry (MBL International, M167-11) overnight at 4 °C. For TUBE immunoprecipitation, 2 mg of total protein were incubated with 20 μl of 50% control (LifeSensors, UM400) or TUBE1 agarose beads (LifeSensors, UM401). Following incubation, beads were separated by magnet and washed 1× with lysis buffer and 2× with wash buffer (150 mM NaCl, 50 mM Tris pH 7.5, 1x Halt protease and phosphatase inhibitor cocktail). Samples were eluted from magnetic beads by boiling in 1x LDS NuPAGE sample buffer with 1x NuPAGE reducing agent at 70 °C for 10 min. Samples were analyzed by immunoblot as described before.

### MS

For IP/MS experiments, IPs were performed as before, and following washing steps with lysis/wash buffer, samples were prepared for MS. Immunoprecipitation eluates were reduced with 10 mM tris(2-carboxyethyl)phosphine for 30 min at RT and then alkylated with 15 mM iodoacetamide for 45 min at RT in the dark. Alkylation was quenched by the addition of 10 mM DTT. Proteins were isolated by methanol-chloroform precipitation. The protein pellets were dried and then resuspended in 50 μL 200 mM EPPS pH 8.0. The resuspended protein samples were digested with 2 μg LysC overnight at RT followed by the addition of 0.5 μg Trypsin for 6 h at 37 °C. Protein digests were dried, resuspended in 100 μL 1% formic acid, and desalted using 10-layer C18 Stage-tips before being analyzed by LC-MS.

Data were collected using an Orbitrap Exploris 480 mass spectrometer (Thermo Fisher Scientific) equipped with a FAIMS Pro Interface and coupled with a UltiMate 3000 RSLCnano System. Peptides were separated on an EasySpray ES803a 75 μm inner diameter microcapillary column (Thermo Fisher Scientific). Peptides were separated using a 180 min gradient of 10% to 25% acetonitrile in 1.0% formic acid with a flow rate of 350 nl/min.

Each analysis used a TopN data-dependent method. The FAIMS Pro Interface compensation voltages were set to -50 and -70. The data were acquired using a mass range of *m/z* 380 to 1200, resolution 60,000, AGC target 3 × 10^6^, auto maximum injection time, dynamic exclusion of 30 s, and charge states of 2 to 6. Top N 40 data-dependent MS2 spectra were acquired with a scan range starting at m/z 110, resolution 15,000, isolation window of 1.6 m/z, normalized collision energy set at 30%, AGC target 1 x 10^5^ and the automatic maximum injection time.

### LC-MS data analysis

Proteome Discoverer 2.4 (Thermo Fisher Scientific) was used for .RAW file processing and controlling peptide and protein level false discovery rates, assembling proteins from peptides, and protein quantification from peptides. MS/MS spectra were searched against a Uniprot human database (December 2019) with both the forward and reverse sequences as well as known contaminants such as human keratins. Database search criteria were as follows: tryptic with two missed cleavages, a precursor mass tolerance of 20 ppm, fragment ion mass tolerance of 0.6 Da, static alkylation of cysteine (57.02146 Da), and variable oxidation of methionine (15.99491 Da). Peptides were quantified using the MS1 Area, and peptide abundance values were summed to yield the protein abundance values.

Resulting data were filtered to only include proteins that had a minimum of two unique peptides quantified. Abundances were normalized and scaled using in-house scripts in the R framework. Significant changes comparing the relative protein abundance between samples were assessed by moderated *t* test as implemented in the limma package within the R framework (37). A protein was considered a ‘hit’ if it met our predetermined ‘hit’ threshold of *p*-value < 0.01 and fold change > 2.

### Statistical analysis

Other statistical analyses were conducted using GraphPad Prism (GraphPad Software Inc), and relevant statistical analyses are described in the corresponding figure legends. *p*-values are indicated by ∗ < 0.05, ∗∗ < 0.01, ∗∗∗ < 0.001.

## Data availability

All relevant data are included within this article and supporting information. Plasmids will be made available upon reasonable requests.

## Supporting information

This article has the following supporting information:

Supporting Figs. 1–5 (included in figure PDF)

Supporting Table 1 – CRISPR screen read counts

Supporting Table 2 – IP/MS results

## Conflict of interest

The authors declare that they have no conflicts of interest with the contents of this article.

## Supporting information

Supporting Table 2

Figure S1 **proteasomal, and lysosomal acidification inhibition in TF1 and 32D cells lead to increased CSF2RB reporter levels and endogenous CSF2RB protein levels.** (*A*) Western blots of endogenous CSF2RB and VINCULIN in TF1 Cas9 cells treated with DMSO control, 1 μM MLN7243 (E1 inhibitor), 5 μM MLN4924 (neddylation inhibitor), or 10 μM MG132 for 4 h in 5 ng/ml GM-CSF. (*B*) Western blots for CSF2RB and Vinculin from TF1 Cas9 cells treated with 10 μM Chloroquine, 100 nM Bafilomycin A1, or a DMSO control for 4 h. (*C*) Bar graph showing normalized GFP/mCherry ratio of CSF2RB reporter in 32D Cas9 cells treated with 1 μM MLN7243, 5 μM MLN4924, 10 μM MG132, or a DMSO control for 4 h as measured by flow cytometry. Bars are the mean ± SD normalized to the DMSO sample from three biological replicates. P-values calculated by unpaired Student’s *t* test between DMSO and other conditions. (*D*) Western blots for Csf2rb and Vinculin from 32D Cas9 cells treated with 1 μM MLN7243, 5 μM MLN4924, 10 μM MG132, or a DMSO control for 4 h. (*E*) Bar graph showing normalized GFP/mCherry ratio of CSF2RB reporter in 32D Cas9 cells treated with 10 μM Chloroquine, 100 nM Bafilomycin A1, or a DMSO control for 4 h as measured by flow cytometry. Bars are the mean ± SD normalized to the DMSO sample from three biological replicates. P-values calculated by unpaired Student’s *t* test between DMSO and other conditions. (*F*) Western blots for Csf2rb and Vinculin from 32D Cas9 cells treated with 10 μM Chloroquine, 100 nM Bafilomycin A1, or a DMSO control for 4 h.

Figure S2 **Schematic of protein-reporter-based screening strategy.** Cells expressing the protein reporter are infected with sgRNA library and then cultured under specified conditions before cell sorting by GFP-mCherry ratio with flow cytometry. Sorted cells are then sequenced for sgRNA abundance, and the GFP/mCherry high population is compared to the GFP/mCherry low population.

Figure S3 **Ubiquitin-ligase-specific CSF2RB-reporter CRISPR-Cas9 screens reveal STUB1 as a regulator of CSF2RB protein stability in unstimulated and GM-CSF-stimulated THP1 cells.** (*A*) Volcano plot showing gene-level analysis of CSF2RB reporter-based ubiquitin-ligase-specific CRISPR screen in THP1 cells cultured without GM-CSF. Guide counts were collapsed to gene-level (n=4 guides/gene; two-sided empirical rank-sum test-statistics). (*B*) Volcano plot showing gene-level analysis of CSF2RB reporter-based ubiquitin-ligase-specific CRISPR screen in THP1 cells cultured with 5 ng/ml GM-CSF for 60 min. Guide counts were collapsed to gene-level (n = 4 guides/gene; two-sided empirical rank-sum test-statistics).

Figure S4 ***Stub1* and *Chic2* KO lead to increased total CSF2RB protein levels without increases in *Csf2rb* transcription.** (*A* and *B*) Bar graph showing quantification of western blots for Csf2rb in 32D Cas9 cells with (*A*) sgNT, sgStub1-1, or sgStub1-2 or (*B*) sgNT, sgChic2-1, or sgChic2-2 in 0.01 ng/ml IL-3 shown in Fig. 3D. Bar graphs show mean ± SEM of the Csf2rb/Vinculin ratio in three experimental replicates normalized to the sgNT sample. P-values calculated by unpaired Student’s *t* test between sgNT and other conditions. (*C* and *D*) Bar graph showing relative *Csf2rb* expression in 32D Cas9 cells with (*C*) sgNT, sgStub1-1, or sgStub1-2 or (*D*) sgNT, sgChic2-1, or sgChic2-2 cultured in 0.01 ng/ml IL-3. Bars show mean ± SEM of the relative expression of *Csf2rb*/*Gapdh* across three biological replicates with four technical replicates each. P-values calculated by unpaired Student’s *t* test between sgNT and other conditions.

Figure S5 ***Stub1* and *Chic2* KO lead to increased cell-surface levels of Csf2rb with representative flow plots.** Representative flow plots of experiment in Figure 2I, including sgNT with unstained, IgG1 isotype control, and CD131-PE and sgChic2-1/2 and sgStub1-1/2 with CD131PE.

Figure S6 ***Stub1* and *Chic2* KO do not increase cell-surface Il3ra levels in 32D cells.** Bar graph showing median fluorescence intensity for anti-Il3ra-PE (CD123) in 32D Cas9 cells with sgNT, sgChic2-1/2, or sgStub1-1/2 in 5, 0.1, or 0.01 ng/m IL-3 as measured by flow cytometry. Bars show mean ± SD from three biological replicates. P-values calculated by unpaired Student’s *t* test between sgNT and other conditions at each cytokine concentration.

Figure S7 **IL-3 concentration does not alter the interaction between STUB1-V5 and Chic2.** Western blots of anti-V5 tag immunoprecipitation of STUB1-V5 and whole cell lysate for V5, Chic2, and Vinculin in 32D Cas9 cells with luciferase or STUB1-V5 cultured in 5, 0.1, or 0.01 ng/ml IL-3.

Figure S8 **HSP inhibitors do not alter Csf2rb protein stability.** Western blots of Csf2rb and Vinculin in 32D cas9 cells treated with DMSO, 1μM Apoptazole (Apop.), or 100 nM HSP990 for 4 h.

## References

[1] Sanada M., Suzuki T., Shih L.-Y., Otsu M., Kato M., Yamazaki S. (2009). Gain-of-function of mutated C-CBL tumour suppressor in myeloid neoplasms. Nature.

[2] Nagarajan A., Petersen M.C., Nasiri A.R., Butrico G., Fung A., Ruan H.-B. (2016). MARCH1 regulates insulin sensitivity by controlling cell surface insulin receptor levels. Nat. Commun..

[3] Broughton S.E., Dhagat U., Hercus T.R., Nero T.L., Grimbaldeston M.A., Bonder C.S. (2012). The GM–CSF/IL-3/IL-5 cytokine receptor family: From ligand recognition to initiation of signaling. Immunol. Rev..

[4] Broughton S.E., Nero T.L., Dhagat U., Kan W.L., Hercus T.R., Tvorogov D. (2015). The Beta c receptor family - structural insights and their functional implications. Cytokine.

[5] Lanza F., Castagnari B., Rigolin G., Moretti S., Latorraca A., Ferrari L. (1997). Flow cytometry measurement of GM-CSF receptors in acute leukemic blasts, and normal hemopoietic cells. Leukemia.

[6] Budel L.M., Touw I.P., Delwel R., Clark S.C., Lowenberg B. (1989). Interleukin-3 and granulocyte-monocyte colony-stimulating factor receptors on human acute myelocytic leukemia cells and relationship to the proliferative response. Blood.

[7] Park L.S., Waldron P.E., Friend D., Sassenfeld H.M., Price V., Anderson D. (1989). Interleukin-3, GM-CSF, and G-CSF receptor expression on cell lines and primary leukemia cells: Receptor heterogeneity and relationship to growth factor responsiveness. Blood.

[8] Powell J.A., Thomas D., Barry E.F., Kok C.H., McClure B.J., Tsykin A. (2009). Expression profiling of a hemopoietic cell survival transcriptome implicates osteopontin as a functional prognostic factor in AML. Blood.

[9] Watanabe-Smith K., Tognon C., Tyner J.W., Meijerink J.P.P., Druker B.J., Agarwal A. (2016). Discovery and functional characterization of a germline, CSF2RB-activating mutation in leukemia. Leukemia.

[10] Martinez-Moczygemba M., Huston D.P. (2001). Proteasomal regulation of Beta C signaling reveals a novel mechanism for cytokine receptor heterotypic desensitization. J. Clin. Investig..

[11] Yoshimura A., Ohkubo T., Kiguchi T., Jenkins N.A., Gilbert D.J., Copeland N.G. (1995). A novel cytokine-inducible gene CIS encodes an SH2-containing protein that binds to tyrosine-phosphorylated interleukin 3 and erythropoietin receptors. EMBO J..

[12] Sievers Q.L., Petzold G., Bunker R.D., Renneville A., Słabicki M., Liddicoat B.J. (2018). Defining the human C2H2 zinc finger degrome targeted by thalidomide analogs through CRBN. Science.

[13] Sievers Q.L., Gasser J.A., Cowley G.S., Fischer E.S., Ebert B.L. (2018). Genome-wide screen identifies cullin-RING ligase machinery required for lenalidomide-dependent CRL4CRBN activity. Blood.

[14] Słabicki M., Kozicka Z., Petzold G., Li Y.-D., Manojkumar M., Bunker R. (2020). The CDK inhibitor CR8 acts as a molecular glue degrader that depletes cyclin K. Nature.

[15] Connell P., Ballinger C.A., Jiang J., Wu Y., Thompson L.J., Höhfeld J. (2001). The co-chaperone CHIP regulates protein triage decisions mediated by heat-shock proteins. Nat. Cell Biol.

[16] Tsherniak A., Vazquez F., Montgomery P.G., Weir B.A., Kryukov G., Cowley G.S. (2017). Defining a cancer dependency map. Cell.

[17] Wang T., Yu H., Hughes N.W., Liu B., Kendirli A., Klein K. (2017). Gene essentiality profiling reveals gene networks and synthetic lethal interactions with oncogenic ras. Cell.

[18] McFarland J.M., Ho Z.V., Kugener G., Dempster J.M., Montgomery P.G., Bryan J.G. (2018). Improved estimation of cancer dependencies from large-scale RNAi screens using model-based normalization and data integration. Nat. Commun..

[19] Thul P.J., Åkesson L., Wiking M., Mahdessian D., Geladaki A., Blal H.A. (2017). A subcellular map of the human proteome. Science.

[20] Tawo R., Pokrzywa W., Kevei É., Akyuz M.E., Balaji V., Adrian S. (2017). The ubiquitin ligase CHIP integrates proteostasis and aging by regulation of insulin receptor turnover. Cell.

[21] Ravalin M., Theofilas P., Basu K., Opoku-Nsiah K.A., Assimon V.A., Medina-Cleghorn D. (2019). Specificity for latent C termini links the E3 ubiquitin ligase CHIP to caspases. Nat. Chem. Biol..

[22] Sarkar S., Brautigan D.L., Parsons S.J., Larner J.M. (2014). Androgen receptor degradation by the E3 ligase CHIP modulates mitotic arrest in prostate cancer cells. Oncogene.

[23] Cardozo C.P., Michaud C., Ost M.C., Fliss A.E., Yang E., Patterson C. (2003). C-terminal Hsp-interacting protein slows androgen receptor synthesis and reduces its rate of degradation. Arch. Biochem. Biophys..

[24] Liu C., Lou W., Yang J.C., Liu L., Armstrong C.M., Lombard A.P. (2018). Proteostasis by STUB1/HSP70 complex controls sensitivity to androgen receptor targeted therapy in advanced prostate cancer. Nat. Commun..

[25] Fan M., Park A., Nephew K.P. (2005). CHIP (carboxyl terminus of Hsc70-interacting protein) promotes basal and geldanamycin-induced degradation of estrogen receptor-α. Mol. Endocrinol..

[26] Xu W., Marcu M., Yuan X., Mimnaugh E., Patterson C., Neckers L. (2002). Chaperone-dependent E3 ubiquitin ligase CHIP mediates a degradative pathway for c-ErbB2/Neu. Proc. Natl. Acad Sci.

[27] Wang T., Yang J., Xu J., Li J., Cao Z., Zhou L. (2014). CHIP is a novel tumor suppressor in pancreatic cancer and inhibits tumor growth through targeting EGFR. Oncotarget.

[28] Jang K.W., Lee J.E., Kim S.Y., Kang M.-W., Na M.H., Lee C.-S. (2011). The C-terminus of Hsp70-interacting protein promotes met receptor degradation. J. Thorac. Oncol..

[29] Wei Q., Sha Y., Bhattacharya A., Fattah E.A., Bonilla D., Jyothula S.S. (2013). Regulation of IL-4 receptor signaling by STUB1 in lung inflammation. Am. J. Respir. Crit. Care Med..

[30] Afrazi A., Sodhi C.P., Good M., Jia H., Siggers R., Yazji I. (2012). Intracellular heat shock protein-70 negatively regulates TLR4 signaling in the newborn intestinal epithelium. J. Immunol..

[31] Faresse N., Ruffieux-Daidie D., Salamin M., Gomez-Sanchez C.E., Staub O. (2010). Mineralocorticoid receptor degradation is promoted by Hsp90 inhibition and the ubiquitin-protein ligase CHIP. Am. J. Physiol-renal..

[32] Meacham G.C., Patterson C., Zhang W., Younger J.M., Cyr D.M. (2001). The Hsc70 co-chaperone CHIP targets immature CFTR for proteasomal degradation. Nat. Cell Biol.

[33] Ballinger C.A., Connell P., Wu Y., Hu Z., Thompson L.J., Yin L.-Y. (1999). Identification of CHIP, a novel tetratricopeptide repeat-containing protein that interacts with heat shock proteins and negatively regulates chaperone functions. Mol. Cell Biol.

[34] Jiang J., Ballinger C.A., Wu Y., Dai Q., Cyr D.M., Höhfeld J. (2001). CHIP is a U-box-dependent E3 ubiquitin ligase. J. Biol. Chem..

[35] Braekeleer E.D., Douet-Guilbert N., Morel F., Bris M.-J.L., Basinko A., Braekeleer M.D. (2012). ETV6 fusion genes in hematological malignancies: a review. Leuk. Res.

[36] Ran F.A., Hsu P.D., Wright J., Agarwala V., Scott D.A., Zhang F. (2013). Genome engineering using the CRISPR-Cas9 system. Nat. Protoc..

[37] Ritchie M.E., Phipson B., Wu D., Hu Y., Law C.W., Shi W. (2015). Limma powers differential expression analyses for RNA-sequencing and microarray studies. Nucleic Acids Res..

