# Supplementary figures and images for: A STUB1 ubiquitin ligase/CHIC2 protein complex negatively regulates the IL-3, IL-5, and GM-CSF cytokine receptor common β chain (CSF2RB) protein stability
Source: J Biol Chem. 2022 Sep 13;298(10):102484. doi: 10.1016/j.jbc.2022.102484 (PMC9574515; doi:10.1016/j.jbc.2022.102484)

**A.**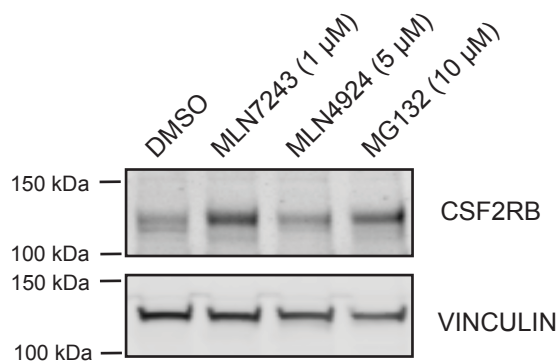**B.**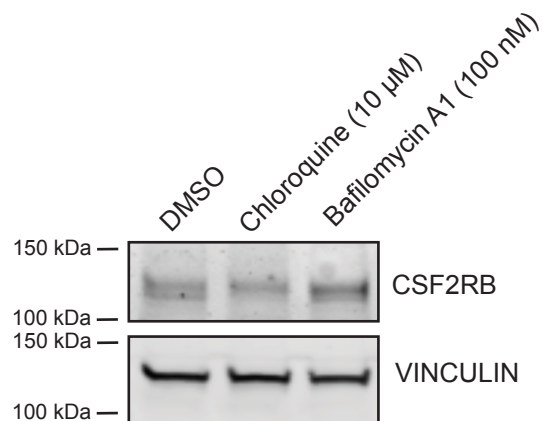**C.**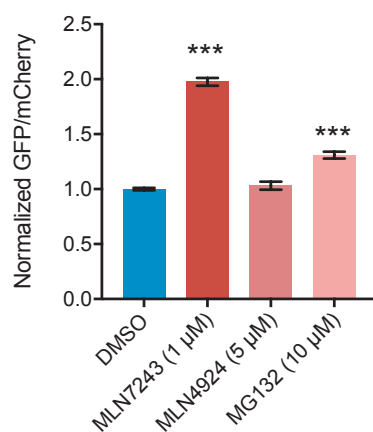**D.**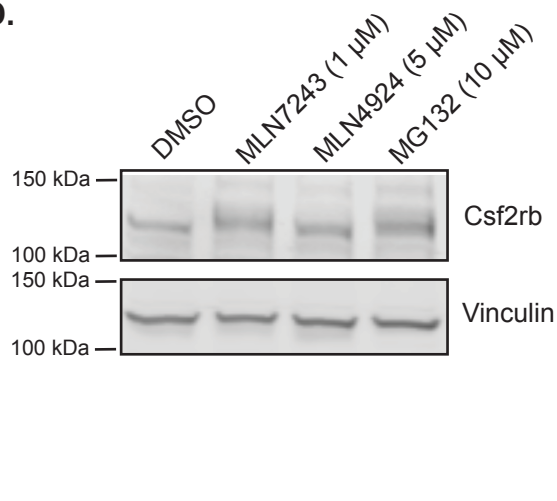**E.**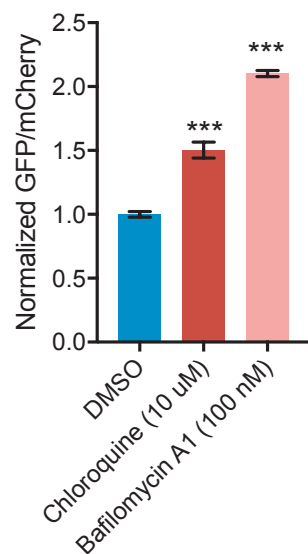**F.**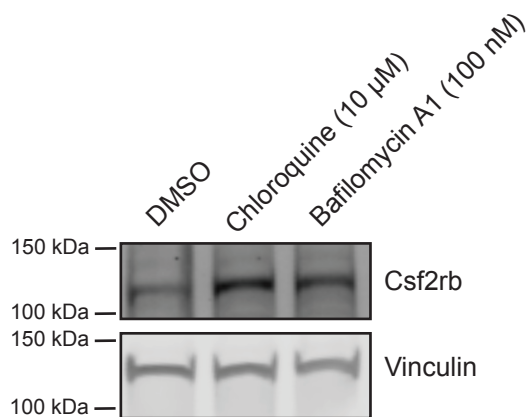

Supplement: Figure S1 — proteasomal, and lysosomal acidification inhibition in TF1 and 32D cells lead to increased CSF2RB reporter levels and endogenous CSF2RB protein levels. (A) Western blots of endogenous CSF2RB and VINCULIN in TF1 Cas9 cells treated with DMSO control, 1 μM MLN7243 (E1 inhibitor), 5 μM MLN4924 (neddylation inhibitor), or 10 μM MG132 for 4 h in 5 ng/ml GM-CSF. (B) Western blots for CSF2RB and Vinculin from TF1 Cas9 cells treated with 10 μM Chloroquine, 100 nM Bafilomycin A1, or a DMSO control for 4 h. (C) Bar graph showing normalized GFP/mCherry ratio of CSF2RB reporter in 32D Cas9 cells treated with 1 μM MLN7243, 5 μM MLN4924, 10 μM MG132, or a DMSO control for 4 h as measured by flow cytometry. Bars are the mean ± SD normalized to the DMSO sample from three biological replicates. P-values calculated by unpaired Student’s t test between DMSO and other conditions. (D) Western blots for Csf2rb and Vinculin from 32D Cas9 cells treated with 1 μM MLN7243, 5 μM MLN4924, 10 μM MG132, or a DMSO control for 4 h. (E) Bar graph showing normalized GFP/mCherry ratio of CSF2RB reporter in 32D Cas9 cells treated with 10 μM Chloroquine, 100 nM Bafilomycin A1, or a DMSO control for 4 h as measured by flow cytometry. Bars are the mean ± SD normalized to the DMSO sample from three biological replicates. P-values calculated by unpaired Student’s t test between DMSO and other conditions. (F) Western blots for Csf2rb and Vinculin from 32D Cas9 cells treated with 10 μM Chloroquine, 100 nM Bafilomycin A1, or a DMSO control for 4 h. [file mmc3.pdf]

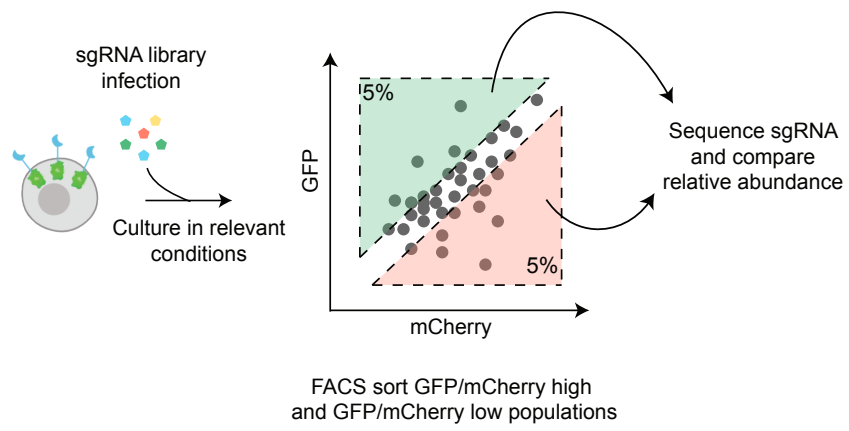

Supplement: Figure S2 — Schematic of protein-reporter-based screening strategy. Cells expressing the protein reporter are infected with sgRNA library and then cultured under specified conditions before cell sorting by GFP-mCherry ratio with flow cytometry. Sorted cells are then sequenced for sgRNA abundance, and the GFP/mCherry high population is compared to the GFP/mCherry low population. [file mmc4.pdf]

**A.**

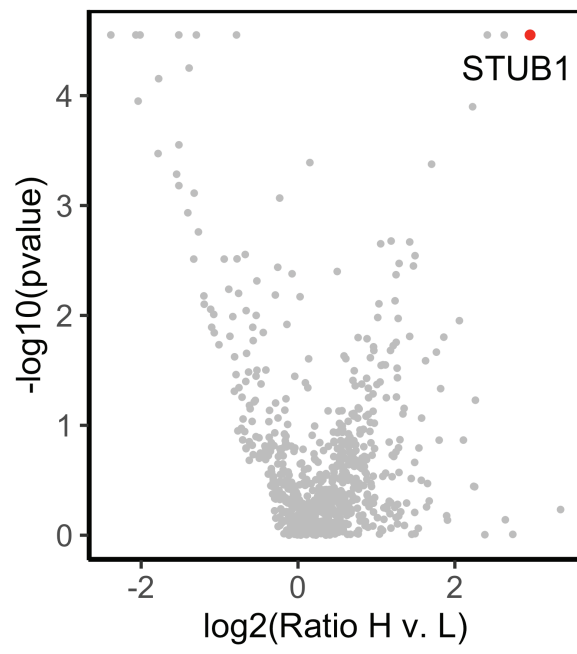

**B.**

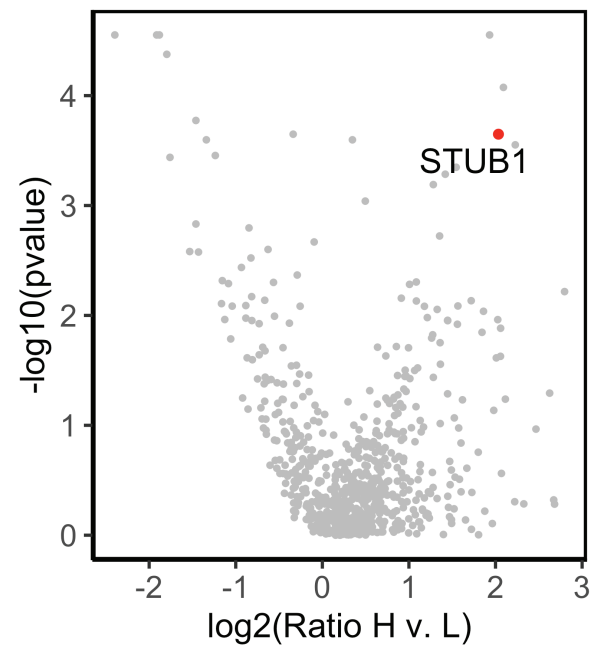

Supplement: Figure S3 — Ubiquitin-ligase-specific CSF2RB-reporter CRISPR-Cas9 screens reveal STUB1 as a regulator of CSF2RB protein stability in unstimulated and GM-CSF-stimulated THP1 cells. (A) Volcano plot showing gene-level analysis of CSF2RB reporter-based ubiquitin-ligase-specific CRISPR screen in THP1 cells cultured without GM-CSF. Guide counts were collapsed to gene-level (n=4 guides/gene; two-sided empirical rank-sum test-statistics). (B) Volcano plot showing gene-level analysis of CSF2RB reporter-based ubiquitin-ligase-specific CRISPR screen in THP1 cells cultured with 5 ng/ml GM-CSF for 60 min. Guide counts were collapsed to gene-level (n = 4 guides/gene; two-sided empirical rank-sum test-statistics). [file mmc5.pdf]

**A.**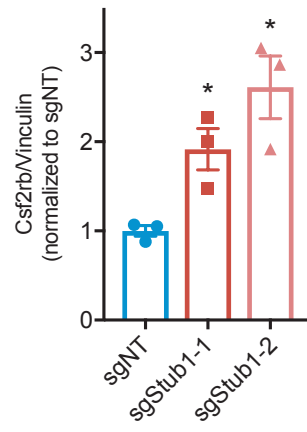**B.**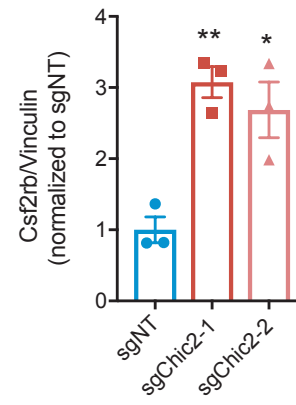**C.**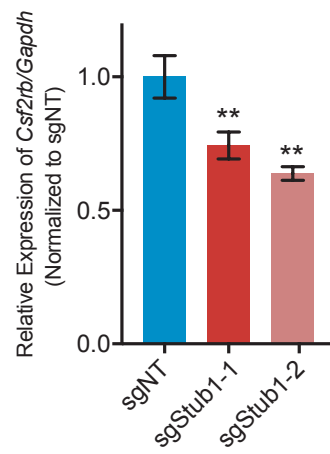**D.**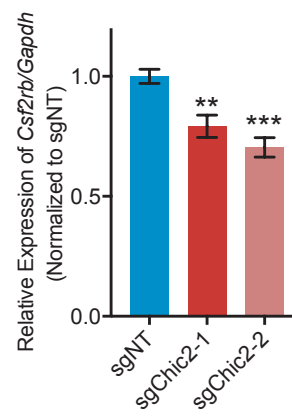

Supplement: Figure S4 — Stub1 and Chic2 KO lead to increased total CSF2RB protein levels without increases in Csf2rb transcription. (A and B) Bar graph showing quantification of western blots for Csf2rb in 32D Cas9 cells with (A) sgNT, sgStub1-1, or sgStub1-2 or (B) sgNT, sgChic2-1, or sgChic2-2 in 0.01 ng/ml IL-3 shown in Fig. 3D. Bar graphs show mean ± SEM of the Csf2rb/Vinculin ratio in three experimental replicates normalized to the sgNT sample. P-values calculated by unpaired Student’s t test between sgNT and other conditions. (C and D) Bar graph showing relative Csf2rb expression in 32D Cas9 cells with (C) sgNT, sgStub1-1, or sgStub1-2 or (D) sgNT, sgChic2-1, or sgChic2-2 cultured in 0.01 ng/ml IL-3. Bars show mean ± SEM of the relative expression of Csf2rb/Gapdh across three biological replicates with four technical replicates each. P-values calculated by unpaired Student’s t test between sgNT and other conditions. [file mmc6.pdf]

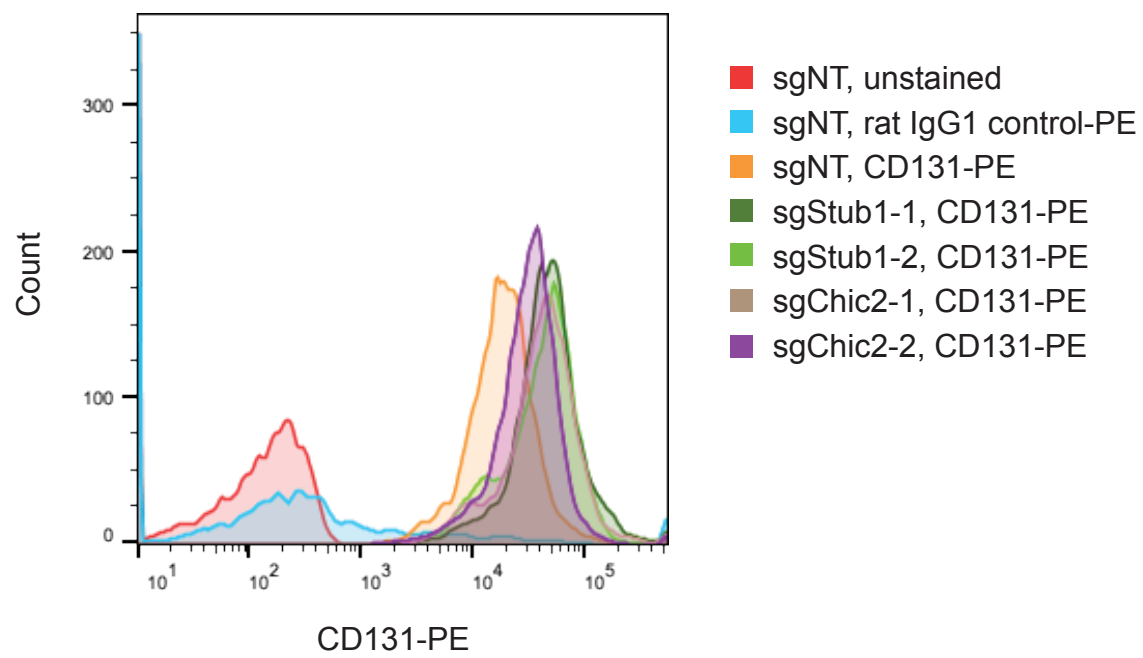

Supplement: Figure S5 — Stub1 and Chic2 KO lead to increased cell-surface levels of Csf2rb with representative flow plots. Representative flow plots of experiment in Figure 2I, including sgNT with unstained, IgG1 isotype control, and CD131-PE and sgChic2-1/2 and sgStub1-1/2 with CD131PE. [file mmc7.pdf]

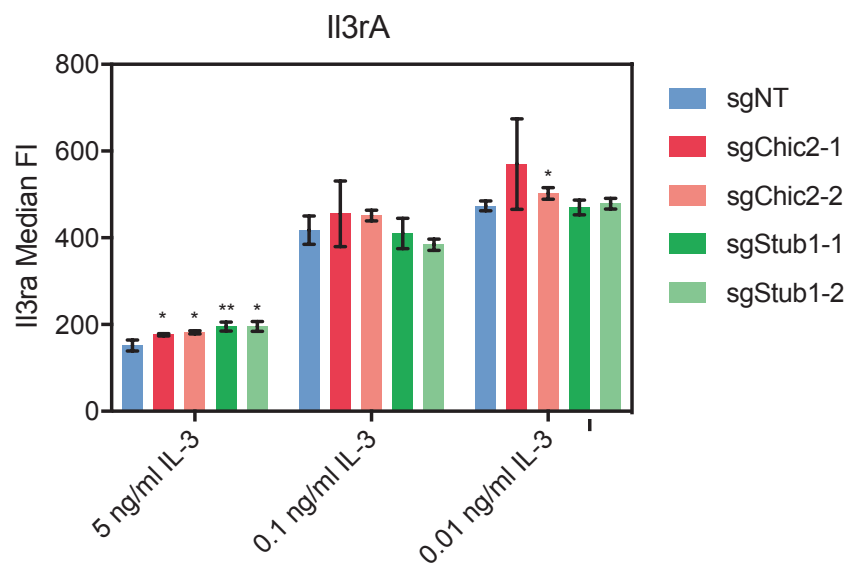

Supplement: Figure S6 — Stub1 and Chic2 KO do not increase cell-surface Il3ra levels in 32D cells. Bar graph showing median fluorescence intensity for anti-Il3ra-PE (CD123) in 32D Cas9 cells with sgNT, sgChic2-1/2, or sgStub1-1/2 in 5, 0.1, or 0.01 ng/m IL-3 as measured by flow cytometry. Bars show mean ± SD from three biological replicates. P-values calculated by unpaired Student’s t test between sgNT and other conditions at each cytokine concentration. [file mmc8.pdf]

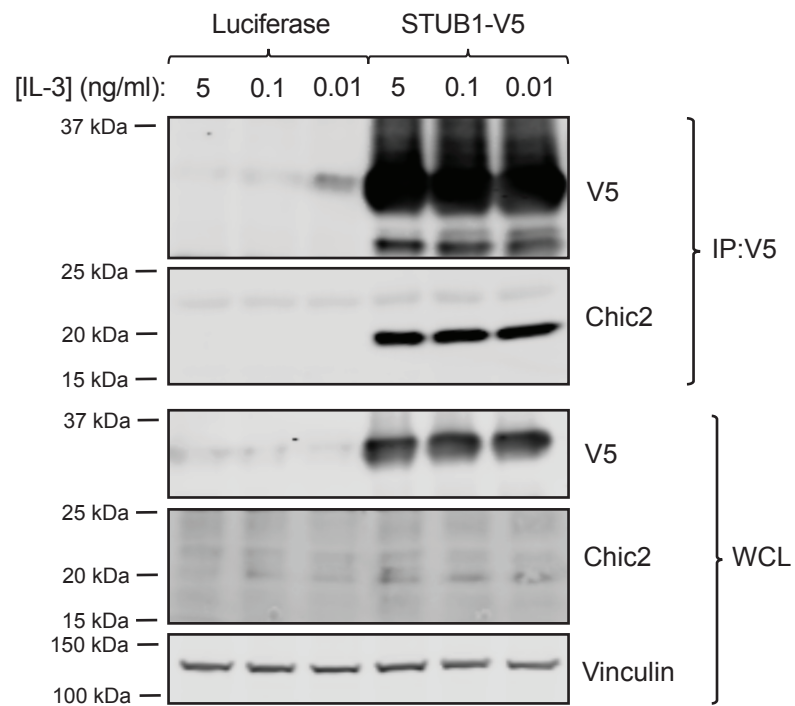

Supplement: Figure S7 — IL-3 concentration does not alter the interaction between STUB1-V5 and Chic2. Western blots of anti-V5 tag immunoprecipitation of STUB1-V5 and whole cell lysate for V5, Chic2, and Vinculin in 32D Cas9 cells with luciferase or STUB1-V5 cultured in 5, 0.1, or 0.01 ng/ml IL-3. [file mmc9.pdf]

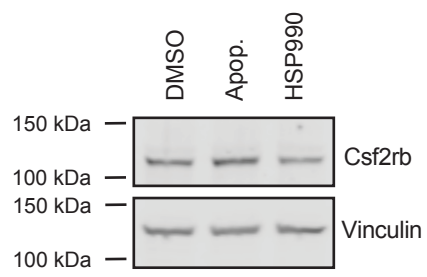

Supplement: Figure S8 — HSP inhibitors do not alter Csf2rb protein stability. Western blots of Csf2rb and Vinculin in 32D cas9 cells treated with DMSO, 1μM Apoptazole (Apop.), or 100 nM HSP990 for 4 h. [file mmc10.pdf]
